# *N*‐oxide‐Functionalized Bipyridines as Strong Electron‐Deficient Units to Construct High‐Performance n‐Type Conjugated Polymers

**DOI:** 10.1002/advs.202414059

**Published:** 2025-01-13

**Authors:** Mingwei Li, Wenhao Li, Junkang Zhou, Xiaowen Tian, Hongxiang Li, Zhen Jiang, Di Liu, Yunqi Liu, Yang Wang, Yongqiang Shi

**Affiliations:** ^1^ Key Laboratory of Functional Molecular Solids Ministry of Education School of Chemistry and Materials Science Anhui Normal University No.189 Jiuhua South Road Wuhu Anhui 241002 China; ^2^ Department of Materials Science State Key Laboratory of Molecular Engineering of Polymers Laboratory of Advanced Materials Fudan University 2005 Songhu Road Shanghai 200438 China; ^3^ College of Polymer Science and Engineering State Key Laboratory of Polymer Materials Engineering Sichuan University Chengdu 610065 China

**Keywords:** bipyridine, electron mobility, n‐type polymer semiconductors, organic thin film transistor, sp^2^‐*N* oxide group

## Abstract

Developing low‐cost unipolar n‐type organic thin‐film transistors (OTFTs) is necessary for logic circuits. To achieve this objective, the usage of new electron‐deficient building blocks with simple structure and easy synthetic route is desirable. Among all electron‐deficient building units, *N*‐oxide‐functionalized bipyridines can be prepared through a simple oxidized transformation of bipyridines. However, employing *N*‐oxide‐functionalized bipyridines as the building unit to construct efficient N‐type polymers has been overlooked. This gap strongly encourages us to design and synthesize two new *N*‐oxide building blocks, 5,5'‐dibromo‐[2,2'‐bipyridine] 1‐oxide (BPyO) and 5,5'‐dibromo‐[2,2'‐bipyridine] 1,1'‐dioxide (BPyDO), through the oxidation of sp^2^‐*N* in 2,2ʹ‐bipyridine. The single‐crystal X‐ray diffraction shows that BPyO and BPyDO possess planar structure with strong π‐stacking, which is beneficial for charge transport. Incorporation of these building blocks into acceptor–acceptor backbones leads to two new polymers, namely P(DPP‐BPyO) and P(DPP‐BPyDO). Both P(DPP‐BPyO) and P(DPP‐BPyDO) possess lower frontier molecular orbital energy levels than the non‐oxide polymer P(DPP‐BPy). Consequently, the transition from P(DPP‐BPy) (without oxide group) to P(DPP‐BPyO) (mono‐oxide group) and then to P(DPP‐BPyDO) (dioxide group) can decrease hole‐transport performance and gradually switch the transport nature from p‐type to n‐type via ambipolar. These results prove that the introduction of sp^2^‐*N* oxide groups in building units would be a promising strategy to approach high‐performance n‐type polymers.

## Graphical abstract

Two new acceptors, BPyO and BPyDO, with simple structure and highly electron‐deficient properties are synthesized by oxidation of sp^2^‐*N* in 2,2ʹ‐bipyridine and incorporated into polymer backbone. Consequently, the transition from P(DPP‐BPy) (without oxide group) to P(DPP‐BPyO) (mono‐oxide group) and then to P(DPP‐BPyDO) (dioxide group) led to dramatically diminished p‐type charge‐transport performance accomplished by enhancement of the n‐type one in OTFTs.

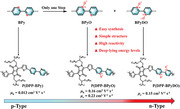

## 1 Introduction

Developing novel π‐conjugated building blocks plays a crucial role in modulating the optoelectronic properties of organic semiconductors. Conjugated polymers with high charge carrier mobility are desirable to approach high device performance, especially for application in organic thin‐film transistors (OTFTs).^[^ ^1^, ^2^, ^3^, ^4^, ^5^, ^6^, ^7^ ^]^ p‐Type (hole‐transporting) polymer semiconductors have exhibited high hole mobility (*µ*_h_) over 10 cm^2^ V^−1^ s^−1^, which is superior to amorphous silicon.^[^ ^8^, ^9^, ^10^ ^]^ However, the development of n‐type (electron‐transporting) polymer semiconductors lags far behind p‐type counterparts.^[^ ^11^, ^12^, ^13^ ^]^ To build high‐performance complementary logic circuits, n‐type and p‐type polymer semiconductors should have balanced electron and hole transport properties.^[^ ^14^, ^15^, ^16^ ^]^ Therefore, more efforts should be devoted to developing n‐type polymer semiconductors.

**Figure 1:**
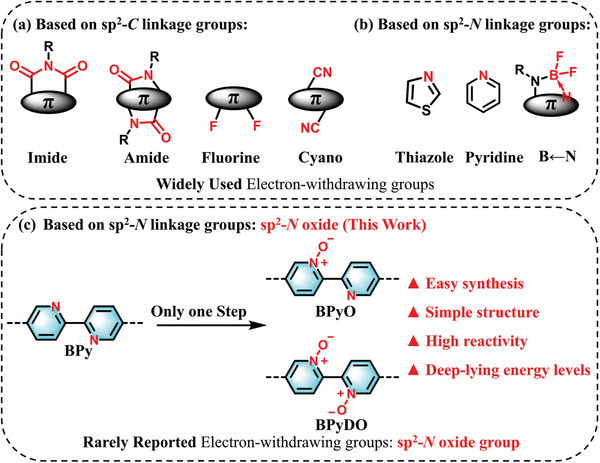
a) Widely used electron‐withdrawing units based on sp^2^‐*C* aromatic system. b) Represented acceptors based on sp^2^‐*N* linkage. c) Rarely reported sp^2^‐*N* oxide groups with strong electron‐withdrawing abilities (This work).

To construct high‐performance n‐type OTFT materials, researchers usually introduce strong electron‐withdrawing groups (EWGs) onto the backbone to lower the lowest unoccupied molecular orbital (LUMO) energy level.^[^ ^17^, ^18^, ^19^, ^20^, ^21^, ^22^ ^]^ Some representative electron‐deficient units based on sp^2^‐*C* aromatic system have been reported and extensively utilized for constructing n‐type polymer semiconductors, such as imide,^[^ ^23^, ^24^, ^25^, ^26^, ^27^ ^]^ amide,^[^ ^28^, ^29^, ^30^ ^]^ halogen atoms (F or Cl),^[^ ^31^, ^32^, ^33^ ^]^ and cyano^[^ ^34^, ^35^, ^36^ ^]^ (**Figure** **1**a). For example, Guo et al. reported a series of imide‐based polymers and showed excellent device performance in OTFTs (*µ*_e_ > 3 cm^2^ V^−1^ s^−1^).^[^ ^37^ ^]^ However, most of these electron‐deficient building blocks based on sp^2^‐*C* aromatic system are obtained with lengthy and complicated synthesis steps, which seriously limits their practical application in OTFTs. Another class of electron‐deficient building blocks are based on sp^2^‐*N* linkage, such as thiazole, pyridine, and boron←nitrogen (B←N) groups (Figure 1b), which have been widely studied in OTFTs, organic thermoelectrics (OTEs) and organic solar cells.^[^ ^38^, ^39^, ^40^, ^41^, ^42^ ^]^ For example, Liu et al. developed a series of polymers based on double B←N bridged bipyridine electron‐deficient building block (BNBP), displaying excellent n‐type performance in OTFTs and OTEs.^[^ ^43^ ^]^

**Figure 2:**
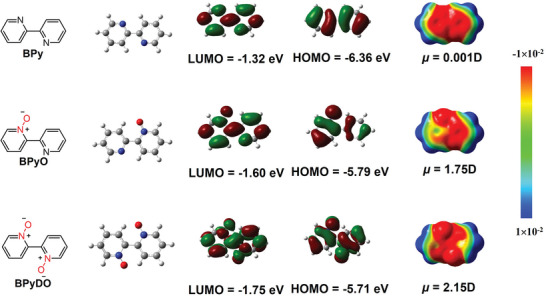
Chemical structures, optimized geometries, theoretical molecular energy levels (calculations are carried out at the B3LYP/6‐31G (d) level), and electrostatic surface potential (ESP) for BPy, BPyO, and BPyDO.

During the exploration of various electron‐deficient building blocks for the synthesis of n‐type polymer semiconductors, we became interested in these sp^2^‐*N* aromatic structures because they can be further oxidized into *N*‐oxide compounds, which can afford even lower LUMO energy levels.^[^ ^44^, ^45^, ^46^ ^]^ Therefore, high electron‐transporting materials might be achieved by introducing *N*‐oxide group into the conjugated backbone. However, less attention has been paid to these *N*‐oxide‐containing conjugated polymers for application in organic electronics. Herein, we designed and synthesized two novel electron‐deficient building blocks containing the *N*‐oxide group, namely 5,5'‐dibromo‐[2,2'‐bipyridine] 1‐oxide (BPyO) and 5,5'‐dibromo‐[2,2'‐bipyridine] 1,1'‐dioxide (BPyDO) (Figure 1c). BPyO and BPyDO possess simple molecular structures and can be synthesized in only one step reaction from cheap raw materials. Compared to non‐*N*‐oxide analogous monomer bipyridine (BPy), BPyO and BPyDO show lower frontier molecular orbital (FMO) energy levels, which is due to the more electron‐withdrawing *N*‐oxide group. Such structurally‐simple and easily accessible strong electron‐deficient building blocks are highly pursued for developing n‐type organic semiconductors. Density functional theory (DFT) calculations of *N*‐oxide‐functionalized bipyridine demonstrate that sp^2^‐*N* oxide groups can effectively reduce the LUMO energy levels. Electrostatic surface potential (ESP) shows more negative potential at N^+^─O^−^ group and large molecular dipole moment (*µ*) in BPyO and BPyDO (**Figure** **2**). Using BPyO and BPyDO as building units, two acceptor–acceptor polymers (P(DPP‐BPyO) and P(DPP‐BPyDO)) are successfully developed. P(DPP‐BPyDO) possesses lower FMO energy levels than the reference polymer P(DPP‐BPy), which can facilitate electron injection and transport in OTFTs. Consequently, the non‐*N*‐oxide polymer P(DPP‐BPy) exhibits unipolar hole transport properties with *µ*_h_ = 0.013 cm^2^ V^−1^ s^−1^, while the mono‐oxide polymer P(DPP‐BPyO) exhibits n‐type dominated ambipolar transport properties with *µ*_h_ = 0.16 cm^2^ V^−1^ s^−1^ and *µ*_e_ = 0.23 cm^2^ V^−1^ s^−1^, respectively. To our delight, the dioxide polymer P(DPP‐BPyDO) shows unipolar electron transport properties with *µ*_e_ = 0.15 cm^2^ V^−1^ s^−1^. We find that the introduction of sp^2^‐*N* oxide group on bipyridine makes unipolar p‐type polymer (P(DPP‐BPy)) switch to unipolar n‐type polymer (P(DPP‐BPyDO)). To the best of our knowledge, this is the first report of unipolar n‐type OTFTs based on N^+^─O^−^ functionalized conjugated polymers. The switched charge carrier polarity from p‐type (P(DPP‐BPy)) to n‐type (P(DPP‐BPyDO)) is benefits from the lowered LUMO level that facilitates electron injection. This study indicates that bipyridine *N*‐oxide‐containing polymers represent a new class of unexplored conjugated polymers with the potential for unique physical and electronic properties.

## 2 Results and Discussion

### 2.1 Synthesis of the *N*‐oxide Monomers and Polymers

**Scheme 1:**
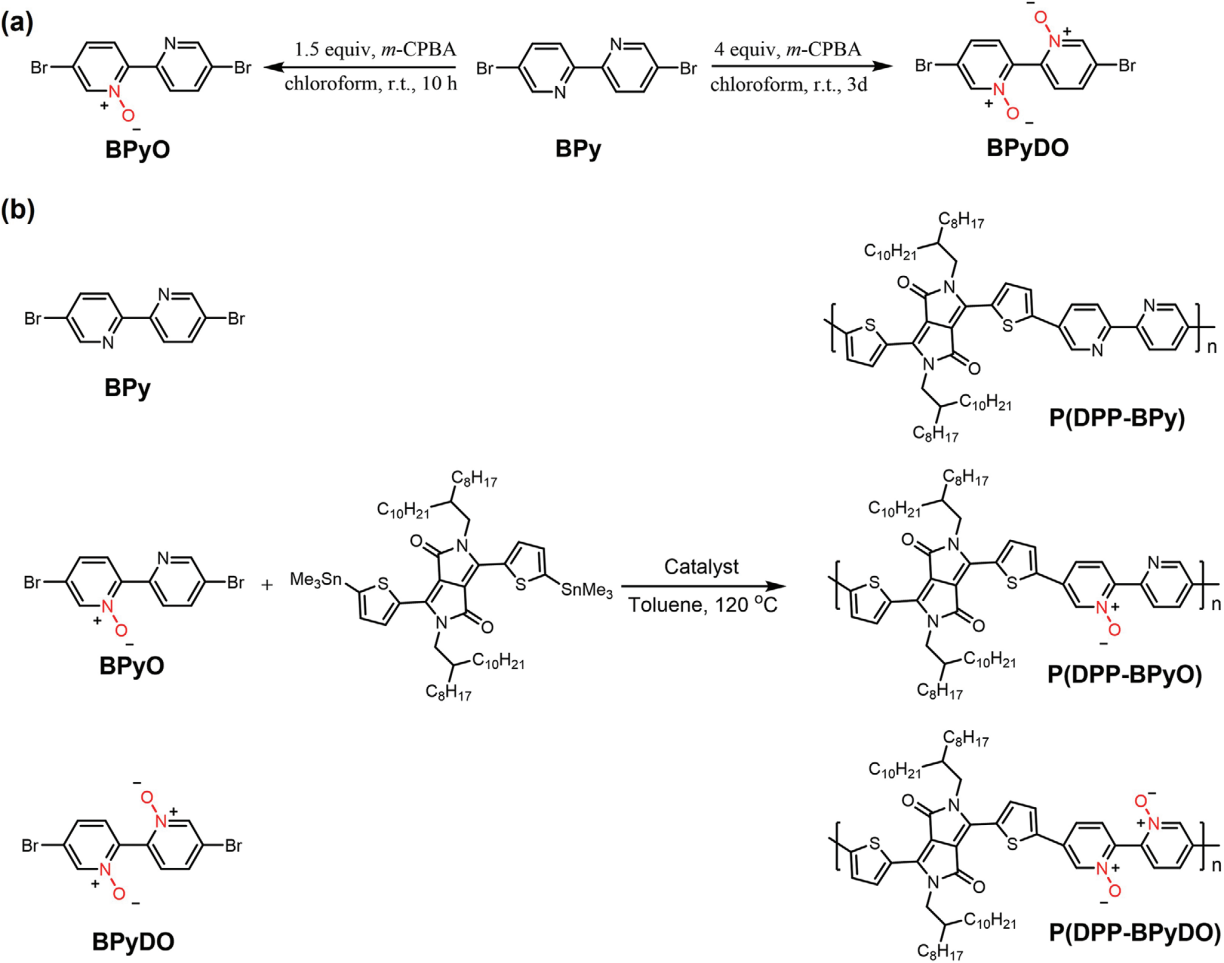
(a) Synthetic route to *N*‐oxide monomers BPyO and BPyDO. (b) Acceptor‐acceptor type polymers P(DPP‐BPy), P(DPP‐BPyO), and P(DPP‐BPyDO).

The synthetic routes to monomers BPyO and BPyDO are shown in Scheme 1a, and the details are provided in the Supporting Information. Attaching oxygen atoms to bipyridine to form mono‐*N*‐oxide bipyridine (BPyO) or *N,Nʹ*‐dioxide bipyridine (BPyDO) can be easily realized. With 1.5 equiv. *m*‐chloroperbenzoic acid (*m*‐CPBA) as the oxidant and chloroform as the solvent, the corresponding BPyO was afforded in 89% yield after stirring for 10 h at room temperature. Bipyridine *N,Nʹ*‐dioxide BPyDO was obtained through oxidation of the BPy with 4.0 equiv. *m*‐CPBA for 3 days. After reaction, the resulting product was purified through recrystallization to yield BPyDO with 67% yield. It takes only one step to synthesize BPyO and BPyDO monomers from commercially available starting materials under mild conditions, which is much easier than other electron‐deficient building blocks with long synthetic steps and multiple purifications. The chemical structures of BPyO and BPyDO were unambiguously identified by ^1^H NMR, ^13^C NMR spectroscopy and high‐resolution mass spectrometry (HR‐MS) (Figures S2–S7, Supporting Information).

Monomers BPyO and BPyDO were copolymerized with distannylated DPP monomer under typical Stille coupling conditions. For comparison, we also synthesized P(DPP‐BPy) without *N*‐oxidation under the same polymerization conditions. Unfortunately, almost no product was obtained from the reaction between BPy and DPP with Pd_2_(dba)_3_/P(*o*‐tolyl)_3_ as catalyst system (Figure S1, Supporting Information). This result was likely due to the diminished reactivity of the bipyridine monomer, potentially a consequence of the chelation between the Pd catalyst and the nitrogen atoms within the bipyridine moieties. Subsequently, copper iodide (CuI) was tried as a cocatalyst because it reacts with organostannanes to produce transient organic copper intermediates and accelerate the transmetallation reaction in the catalytic cycle. This was observed to significantly enhance the polymerization process, leading to the successful synthesis of P(DPP‐BPy) with a number‐average molecular weight (*M*_n_) of 8.0 kDa.^[^ ^21^ ^]^ However, under the same catalyst conditions, P(DPP‐BPyO) and P(DPP‐BPyDO) still exhibit ideal *M*_n_ values without the cocatalyst CuI. This suggests that both mono *N*‐oxide and *N,N'*‐dioxide groups effectively block the coordination of bipyridine. Further optimization of these polymerization conditions was pursued using Pd(PPh_3_)_4_ as a catalyst, a higher *M*_n_ of 22.5 kDa and a polydispersity index (PDI) of 1.5 were achieved for P(DPP‐BPy). This result demonstrated that the absence of chelation between Pd(PPh_3_)_4_ and BPy monomer, which is likely attributable to the distinct catalytic mechanisms of the two Pd catalysts for Stille coupling. The above polymerization conditions were also used to synthesize P(DPP‐BPyO) and P(DPP‐BPyDO). It is worth noting that the reaction rate between BPyO/BPyDO and DPP is surprisingly fast, evidenced by the color of the reaction solution that switches from red to deep green in 10 min after the reaction starts (Figure S1, Supporting Information). Compared to P(DPP‐BPy), both P(DPP‐BPyO) and P(DPP‐BPyDO) exhibit ideal *M*_n_s as presented in **Table** **1**, indicating the successful modification of the polymer backbone. This modification appears to have dual benefits. It not only reduces the molecular orbital energy level to facilitate electron injection but also blocks the coordination between bipyridine and catalyst, thereby improving the reactivity of monomers. These findings underscore the potential of *N*‐oxide‐containing conjugated polymers for applications in organic electronic fields. The ability to achieve both improved electronic properties and enhanced reactivity through a single modification is indeed a significant advancement. These polymers were purified by Soxhlet extraction with methanol, acetone, hexane, and dichloromethane to remove the low‐molecular weight fractions. The final chloroform fraction was collected and concentrated to obtain the desired polymers. The *M*_n_s and PDI of these polymers were evaluated by high‐temperature gel permeation chromatography (HT‐GPC) with 1,2,4‐trichlorobenzene as the eluent at 150 °C (Table 1). These three polymers exhibit excellent thermal stability with 5% mass loss temperature (*T*_d_) over 310 °C (Figure S11a, Supporting Information). The thermal transition of three polymers was recorded in the range from 50 to 250 °C by DSC. No obvious transition peaks were observed, revealing that no phase transitions in this temperature range (Figure S11b, Supporting Information).

**Table 1:** Summary of reaction conditions for the polymers P(DPP‐BPy), P(DPP‐BPyO), and P(DPP‐BPyDO).

Polymer	Catalytic conditions	Time	*M*_n_ [kDa]	PDI	Yield [%]
P[DPP‐BPy]	Pd_2_[dba]_3_ P[*o*‐tol]_3_	3 d	–	–	–
	Pd_2_[dba]_3_ P[*o*‐tol]_3_ CuI	3 d	8.0	2.9	39
	Pd[PPh_3_]_4_	3 d	22.5	1.5	58
P[DPP‐BPyO]	Pd_2_[dba]_3_ P[*o*‐tol]_3_	37 min	49.7	2.1	79
	Pd_2_[dba]_3_ P[*o*‐tol]_3_ CuI	10 h	33.4	1.8	74
	Pd[PPh_3_]_4_	3 d	50.9	2.6	70
P[DPP‐BPyDO]	Pd_2_[dba]_3_ P[*o*‐tol]_3_	3 d	53.3	1.8	75
	Pd_2_[dba]_3_ P[*o*‐tol]_3_ CuI	3 d	30.0	1.7	66
	Pd[PPh_3_]_4_	3 d	35.0	1.6	68

### 2.2 Single Crystal Structures

**Figure 3:**
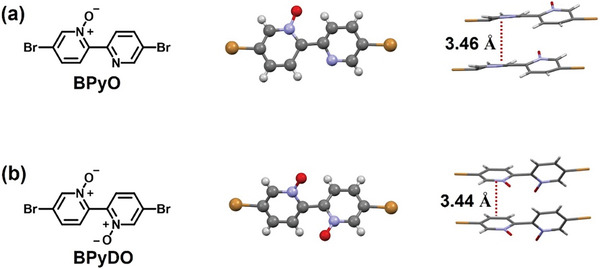
Single crystal structures and packing modes of a) BPyO and b) BPyDO.

Single crystals of *N*‐oxide BPyO and *N,Nʹ*‐dioxide BPyDO were obtained by slow evaporation of dichloromethane solution. As shown in **Figure** **3**, the oxygen atoms were indeed attached onto the nitrogen atoms. In the crystal structures of BPyO and BPyDO, the dihedral angles are slightly off‐planar. The intermolecular π–π stacking distance between the pyridine cores were found to be similar in the two molecules: 3.46 Å in BPyO and 3.44 Å in BPyDO.^[^ ^47^ ^]^ The single crystal features of the *N*‐oxide compounds demonstrate the great potentials of BPyO and BPyDO for constructing polymers with a planar backbone and close π‐stacking distance, which is conducive to efficient charge transport.

### 2.3 Optical and Electrochemical Properties of Polymers

**Figure 4:**
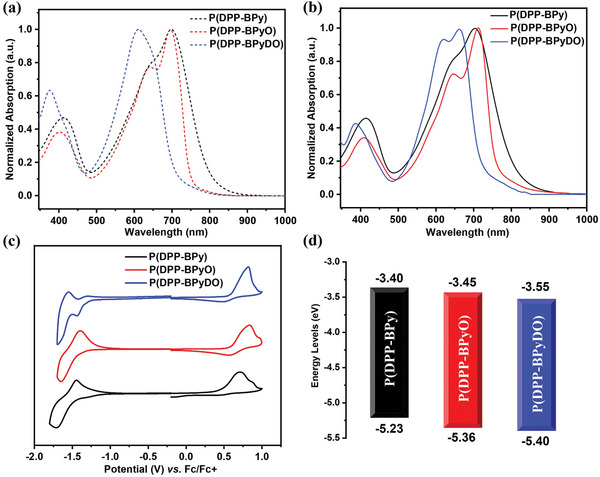
a) Normalized UV–vis absorption spectra of P(DPP‐BPy), P(DPP‐BPyO) and P(DPP‐BPyDO) in chloroform and b) thin‐films. c) Cyclic voltammograms curves of three polymers in film state. d) These polymers FMO energy levels diagram.

To investigate the effect of sp^2^‐*N* oxide groups on the optical properties of these polymers P(DPP‐BPy), P(DPP‐BPyO) and P(DPP‐BPyDO). UV–vis absorption spectra for these polymers were recorded both in chloroform solution and as thin films (**Figure** **4**a,b), and the corresponding absorption parameters are summarized in **Table** **2**. In solution, P(DPP‐BPy) displayed a broad absorption range from 500–800 nm, whereas, P(DPP‐BPyO) and P(DPP‐BPyDO) demonstrated a relatively confined range of 500–750 and 500–700 nm, respectively. Furthermore, the absorption peaks of these polymers showed a red‐shift from the solution to the thin film states, with the magnitude of the red‐shift increasing from P(DPP‐BPy) (6 nm) to P(DPP‐BPyO) (17 nm) and P(DPP‐BPyDO) (52 nm). Additionally, obvious shoulder peaks are observed in the thin film spectra of P(DPP‐BPyO) and P(DPP‐BPyDO), indicating stronger aggregation behavior and enhanced intermolecular interactions in the solid state. These changes can be attributed to the introduction of the sp^2^‐*N* oxide groups, which can facilitate charge transport by enhancing the intermolecular interactions. Upon increasing the level of *N*‐oxidation, a blue‐shift is observed in the onset of film absorption from 832to 775 nm, and to 723 nm for P(DPP‐BPy), P(DPP‐BPyO), and P(DPP‐BPyDO), respectively, corresponding to a decrease in the optical band gap of ≈0.1 eV per each successive *N*‐oxidation. The optical energy band gaps (*E*_g_^opt^) of P(DPP‐BPy), P(DPP‐BPyO), and P(DPP‐BPyDO) are 1.49, 1.60, and 1.70 eV, respectively, calculated from the absorption onset of polymers in solid states. The introduction of the *N*‐oxide groups on the bipyridine units decreases the electron‐donating ability of the bipyridine moiety, thereby reducing the overall push‐pull electron effect within the polymer backbone. This reduction in the push‐pull effect leads to an increase in the *E*_g_^opt^, as observed in the gradual widening of the *E*_g_^opt^ values from P(DPP‐BPy) to P(DPP‐BPyO) and P(DPP‐BPyDO). Overall, the results demonstrate that the introduction of sp^2^‐*N* oxide groups can effectively tune the optical properties of the conjugated polymers, leading to changes in the absorption spectra, aggregation behavior, and energy band gaps, which are crucial for their performance in optoelectronic applications.

**Table 2:** Optical and electrochemical properties of P(DPP‐BPy), P(DPP‐BPyO) and P(DPP‐BPyDO).

Polymer	*λ*_max_^sol^ ^a)^ [nm]	*λ*_max_^film^ ^b)^ [nm]	*λ*_onset_^film^ ^b)^ [nm]	*E*_g_^opt^ ^c)^ [eV]	*E*_LUMO_ ^d)^ [eV]	*E*_HOMO_ ^d)^ [eV]
P[DPP‐BPy]	698	703	832	1.49	−3.40	−5.23
P[DPP‐BPyO]	695	712	775	1.60	−3.45	−5.36
P[DPP‐BPyDO]	611	662	723	1.70	−3.55	−5.40

^a)^ Absorption of chloroform solution (10^−5^ M);

^b)^ Absorption spectra of the pristine film cast from 5 mg mL^−1^ chloroform solution;

^c)^ Estimated from the absorption onset of as‐cast film using the equation *E*_g_^opt^ = 1240/*λ*_onset_ (eV);

^d)^ *E*_HOMO/LUMO_ = ‐e(*E*_ox/red_^onset^ – *E*_Fc/Fc_^+^ + 4.80) eV; *E*_ox/red_^onset^ determined using the Fc/Fc^+^ external standard.

The electrochemical properties of these polymers were evaluated by cyclic voltammetry (CV) to gain insight into their energy levels, using the ferrocene/ferrocenium (Fc/Fc^+^) redox couple as the external standard (Figure 4c). From P(DPP‐BPy) to P(DPP‐BPyO) and then to P(DPP‐BPyDO), the reduction peaks became more prominent while the oxidation peaks gradually weakened, indicating that the introduction of the N^+^─O^−^ group resulted in enhanced n‐type characteristics and diminished p‐type characteristics. The LUMO energy levels of P(DPP‐BPy), P(DPP‐BPyO), and P(DPP‐BPyDO) were determined from the onset potential of the reduction peaks, which were −3.40, −3.45, and −3.55 eV, respectively. Clearly, the LUMO energy level of P(DPP‐BPyDO) decreased after the introduction of the *N,N*′‐dioxide groups compared to that of P(DPP‐BPy). The highest occupied molecular orbital (HOMO) energy levels of P(DPP‐BPy), P(DPP‐BPyO), and P(DPP‐BPyDO) were calculated using the equation: *E*_HOMO_ = ‐e(*E*_ox_^onset^ – *E*_Fc/Fc_^+^ + 4.80) eV, which were −5.23, −5.36, and −5.40 eV, respectively. The results demonstrated that sp^2^‐*N* oxide groups effectively downshifted the FMO energy levels and enhanced electron affinity, which contributed to unipolar n‐type charge transport.

### 2.4 Theoretical Calculations

**Figure 5:**
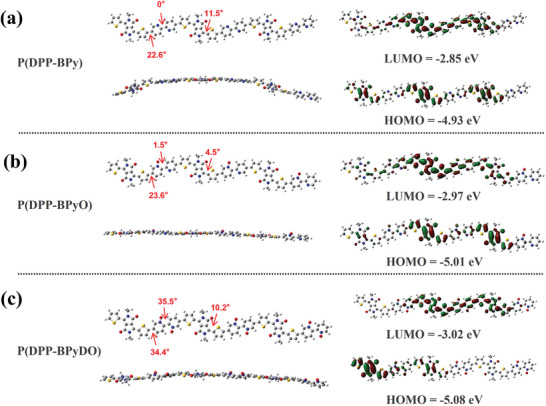
Calculated molecular orbitals of trimers of P(DPP‐BPy), P(DPP‐BPyO), and P(DPP‐BPyDO) at the B3LYP/6‐31G (d) level. All alkyl chains are replaced with methyl chains for computational simplicity.

To further investigate the electronic structure and optimize the conformation of P(DPP‐BPy), P(DPP‐BPyO) and P(DPP‐BPyDO), density functional theory (DFT) calculations were conducted on trimers of the polymer repeating units at B3LYP/6‐31G (d) level. All alkyl side chains were simplified to methyl (**Figure** **5**). Compared to P(DPP‐BPy), P(DPP‐BPyO) and P(DPP‐BPyDO) possess relatively planar backbone. The distances between H and O atoms are 2.05 and 2.16 Å in BPyO and BPyDO units, respectively (Figure 5b,c), which are shorter than the sum of H and O van der Waals radius of 2.60 Å. This indicates the presence of H⋅⋅⋅O noncovalent interaction. The theoretical energy levels gradually decrease from P(DPP‐BPy) to P(DPP‐BPyO) and P(DPP‐BPyDO), consistent with the experimental data. Furthermore, the electrostatic potential (ESP) of these three polymers was investigated (Figure S12, Supporting Information). The oxygen atoms in BPyO and BPyDO units show more negative potential, attributable to the electron‐withdrawing effect of the sp^2^‐*N* oxide groups. The introduction of sp^2^‐*N* oxide groups also enhances the molecular dipole moment (*µ*), P(DPP‐BPyO) and P(DPP‐BPyDO) exhibit larger *µ* values of 5.26 D and 5.23 D, respectively, compared to 0.72D for P(DPP‐BPy). This contributes to stronger molecular interaction and tighter π–π stacking, which are beneficial for charge transport.

### 2.5 Polymer Charge Transport Properties

**Figure 6:**
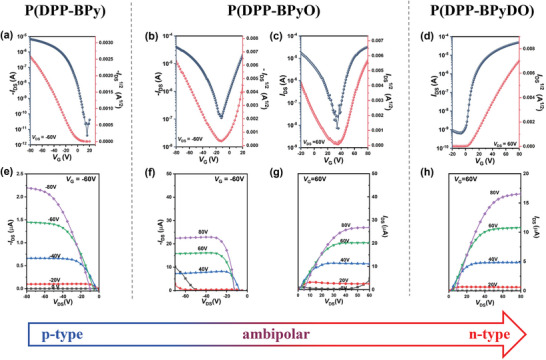
p‐type transfer a,b) and output characteristics e,f) of P(DPP‐BPy) and P(DPP‐BPyO). n‐type transfer c,d) and output characteristics g,h) of P(DPP‐BPyO) and P(DPP‐BPyDO).

The charge transport properties of P(DPP‐BPy), P(DPP‐BPyO), and P(DPP‐BPyDO) were investigated under three catalytic conditions to assess the impact of sp^2^‐*N* oxide groups, using a top‐gate/bottom‐contact (TG/BC) OTFT device structure. The details of device fabrication are provided in the Supporting Information. Notably, the resultant polymers demonstrated optimal performance when Pd(PPh_3_)_4_ was utilized as the catalyst, followed by thermal annealing at 100 °C 30 min. This suggests fewer defects in the polymers obtained under such conditions and an optimized molecular stacking structure after annealing. The device performances of other polymers obtained through different polymerization conditions were also characterized (Figures S13–S15 and Table S2, Supporting Information). As shown in **Figure** **6**, these polymers showed remarkable performance difference in OTFTs. P(DPP‐BPy) displayed unipolar p‐type characteristic with a *µ*_h_ of 0.013 cm^2^ V^−1^ s^−1^ (Figure 6a). With the introduction of mono‐*N*‐oxide group in P(DPP‐BPyO), the LUMO energy level decreased from −3.40 to −3.45 eV compared to non‐oxide P(DPP‐BPy). Consequently, the mono *N*‐oxide P(DPP‐BPyO) exhibited n‐type dominated ambipolar charge transport with hole and electron mobilities of 0.16 and 0.23 cm^2^ V^−1^ s^−1^, respectively. In contrast, *N,Nʹ*‐dioxide P(DPP‐BPyDO) exhibited unipolar n‐type charge transport with a *µ*_e_ of 0.15 cm^2^ V^−1^ s^−1^, a relatively higher on/off current (*I*_on_/*I*_off_) of 10^4^–10^5^ and a low threshold voltage of 5 to 10 V. To the best of our knowledge, this is the first report of unipolar n‐type OTFTs based on N^+^─O^−^ functionalized polymers. The low *V*_th_ of P(DPP‐BPyDO) is attributed to its deep‐lying LUMO enabled by the N^+^─O^−^ groups. The transition from p‐type to ambipolar, and ultimately to unipolar n‐type charge‐transport behavior is primarily due to the introduction of electron‐withdrawing N^+^─O^−^ groups, which electronically deepens the HOMO energy levels and suppresses hole accumulation. Therefore, the design strategy of *N*‐oxide polymers provides valuable insights into the development of n‐type polymer semiconductors.

We conducted a comprehensive investigation on the *M*_n_ of polymers under different polymerization conditions and their OTFT performances. As illustrated in Figures S13–S15 and Table S2 (Supporting Information), P(DPP‐BPy), with an *M*_n_ of 8.0 kDa, exhibited unipolar p‐type characteristics with a *µ*_h_ of 0.01 cm^2^ V^−1^ s^−1^. Upon increasing the *M*_n_ from 8.0 to 22.5 kDa, the *µ*_h_ increased to 0.013 cm^2^ V^−1^ s^−1^. However, for P(DPP‐BPyO) obtained using Pd_2_(dba)_3_ catalyst systems, an increase in *M*_n_ led to a significant decrease in both hole and electron mobilities. Conversely, when the catalyst was changed to Pd(PPh_3_)_4_, P(DPP‐BPyO) with a higher *M*_n_ of 50.9 kDa exhibited slightly higher *µ*_e_ and *µ*_h_, possibly due to fewer defects under this polymer condition. For P(DPP‐BPyDO), with an *M*_n_ of 30 kDa, unipolar n‐type characteristics were observed, with a *µ*_e_ of 0.067 cm^2^ V^−1^ s^−1^. However, increasing the *M*_n_ from 30 to 53 kDa resulted in a decrease in *µ*_e_ to 0.056 cm^2^ V^−1^ s^−1^. Indeed, the results highlight the significance of both polymerization conditions and *M*_n_ in influencing the performance of polymers in OTFTs. The polymerization conditions, including the choice of catalyst and reaction parameters, can have a substantial impact on the structural and electronic properties of the resulting polymers, thereby affecting their charge transport characteristics in OTFT devices. Additionally, the molecular weight of the polymers plays a crucial role in determining their solubility, processability, and charge carrier mobility, all of which are essential factors for OTFT applications. Therefore, optimizing both the polymerization conditions and the molecular weight of the polymers is essential for achieving high‐performance organic electronic devices **Table** **3**.

**Table 3:** Transistors performance parameters of the polymers.

Polymer	p‐Type			n‐Type		
	*µ*_h_ ^a)^ [cm ^2^ V^−1^ s^−1^]	*I*_on_/*I*_off_	*V*_th_^b^	*µ*_e_ ^a)^ [cm ^2^ V^−1^ s^−1^]	*I*_on_/*I*_off_	*V*_th_
P[DPP‐BPy]	0.013 [0.010]	10^4^–10^5^	−5–10	–	–	–
P[DPP‐BPyO]	0.16 [0.077]	10^2^–10^3^	−15–20	0.23 [0.095]	10^2^–10^3^	30–45
P[DPP‐BPyDO]	–	–	–	0.15 [0.071]	10^4^–10^5^	5–10

^a)^ maximum mobilities; the average values are in parentheses (>10 devices).

### 2.6 Film Morphology and Microstructure

**Figure 7:**
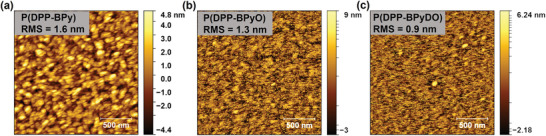
AFM height images of polymer films of a) P(DPP‐BPy), b) P(DPP‐BPyO) and c) P(DPP‐BPyDO).

The thin‐film morphologies of P(DPP‐BPy), P(DPP‐BPyO), and P(DPP‐BPyDO) were investigated by atomic force microscopy (AFM). As depicted in **Figure** **7**, these polymer films exhibited interconnected networks, suggesting the formation of efficient charge‐transport pathways. The root‐mean‐square (RMS) roughness values of these polymers are 1.6, 1.3, and 0.9 nm for P(DPP‐BPy), P(DPP‐BPyO), and P(DPP‐BPyDO), respectively. Notably, these values are relatively low, indicating the formation of the smooth surface in the semiconducting layer. For P(DPP‐BPyDO), the measurement reveals that the films are quite smooth with RMS below 1 nm, indicating weak film crystallinity. In contrast, the P(DPP‐BPyO) film exhibits a larger grain size and higher RMS, likely due to its greater crystallinity, which correlates well with its higher mobility. This suggests that once better controlled film deposition methods are adopted, there is significant potential for further enhancing the performance of OTFTs based on these N^+^─O^−^ polymers.

**Figure 8:**
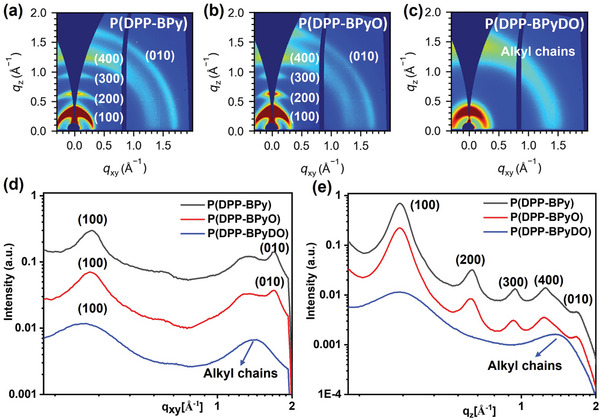
The 2D GIWAXS images of a) P(DPP‐BPy), b) P(DPP‐BPyO), and c) P(DPP‐BPyDO). d) In‐plane and e) out‐of‐plan line‐cut profiles of the corresponding GIWAXS patterns.

To gain insights into the polymer ordering and packing structure, we employed the 2D grazing incidence wide‐angle X‐ray scattering (2D‐GIWAXS) technique. **Figure** **8** shows the 2D‐GIWAXS patterns and corresponding 1D line‐cut profile of the polymer films. P(DPP‐BPy) and P(DPP‐BPyO) films exhibited obvious lamellar diffractions progressing to the fourth order (400) in the out‐of‐plane (OOP) direction (Figure 8a,b). Meanwhile, OOP (010) and in‐plane (IP) (010) peaks can also be observed, suggesting that P(DPP‐BPy) and P(DPP‐BPyO) adopted mixed face‐on and edge‐on orientations in thin films. Based on the OOP (100) and IP (010) diffraction peaks, the lamellar packing and π–π stacking distances were calculated to be 19.39/3.71 Å for P(DPP‐BPy), and 19.41/3.72 Å for P(DPP‐BPyO), respectively. Thus, the incorporation of mono N^+^─O^−^ group does not significantly affect the molecular packing. Therefore, the improvement of the charge mobility of mono‐oxide polymer P(DPP‐BPyO) should be attributed to its deepened FMO levels. However, going from P(DPP‐BPyO) to the *N,N*ʹ‐dioxide polymer P(DPP‐BPyDO), the (h00) diffraction peaks disappeared, implying a largely reduced crystallinity. This reduced polymer packing ordering is not beneficial for charge transport although the dioxide polymer P(DPP‐BPyDO) owns deeper FMO levels. Overall, the higher crystallinity of mono‐oxide polymer with suitable FMO levels P(DPP‐BPyO) is favorable for the hole/electron transport in OTFTs. This result is in good accord with its better OTFT mobility.

## 3 Conclusion

In summary, we have effectively synthesized two innovative electron‐deficient building blocks, BPyO and BPyDO, by incorporating the sp^2^‐*N* oxide groups into the bipyridine backbone. The structures of BPyO and BPyDO were confirmed by single‐crystal X‐ray diffraction. The introduction of sp^2^‐*N* oxide groups onto the backbone not only led to a down‐shift in the FMO energy levels but also enhanced the reactivity of bipyridine. Therefore, two *N‐*oxide polymers, P(DPP‐BPyO) and P(DPP‐BPyDO), with higher molecular weights of >50 kDa, were successfully developed. As a result, the non‐oxide analogous polymer P(DPP‐BPy) exhibited unipolar p‐type transport properties with a hole mobility (*µ*_h_) of 0.013 cm^2^ V^−1^ s^−1^, while the mono *N*‐oxide P(DPP‐BPyO) demonstrated n‐type dominated ambipolar charge transport with hole and electron mobilities of 0.16 and 0.23 cm^2^ V^−1^ s^−1^, respectively. Further switching the charge carrier polarity to pure n‐type was achieved by using *N,Nʹ*‐dioxide polymer P(DPP‐BPyDO) that displayed unipolar n‐type charge transport with an electron mobility (*µ*_e_) of 0.15 cm^2^ V^−1^ s^−1^, a relatively higher on/off current (*I*_on_/*I*_off_) of 10^4^–10^5^ and a low threshold voltage of 5 to 10 V. To the best of our knowledge, this is the first report of unipolar n‐type OTFTs based on the N^+^─O^−^ functionalized polymers. The switched charge carrier polarity demonstrates that introducing the *N*‐oxide group in conjugated polymers is an effective way to enhance the electron transport properties.

## Conflict of Interest

The authors declare no conflict of interest.

## Supporting information



Supporting Information

## Data Availability

The data that support the findings of this study are available in the supplementary material of this article.
