# Supplementary material for: N‐oxide‐Functionalized Bipyridines as Strong Electron‐Deficient Units to Construct High‐Performance n‐Type Conjugated Polymers
Source: Adv Sci (Weinh). 2025 Jan 13;12(9):2414059. doi: 10.1002/advs.202414059 (PMC11884590; doi:10.1002/advs.202414059)
Supplement: Supplementary file 1 — Supporting Information [file ADVS-12-2414059-s001.docx]

**Supporting Information**

**N-oxide-Functionalized Bipyridines as Strong Electron-Deficient Units to Construct High-Performance n-Type Conjugated Polymers**

Mingwei Li,^a,ǁ^ Wenhao Li,^b,ǁ^ Junkang Zhou,^a^ Xiaowen Tian,^a^ Hongxiang Li,^c^ Zhen Jiang,^b^ Di Liu,^b^ Yunqi Liu,^b^ Yang Wang,^b,^* and Yongqiang Shi^a,^*

[a] Key Laboratory of Functional Molecular Solids, Ministry of Education, School of Chemistry and Materials Science, Anhui Normal University, No.189, Jiuhua South Road, Wuhu, Anhui, 241002, China

[b] Department of Materials Science, State Key Laboratory of Molecular Engineering of Polymers, Laboratory of Advanced Materials, Fudan University, 2005, Songhu Road, Shanghai, 200438, China.

[c] College of Polymer Science and Engineering, State Key Laboratory of Polymer Materials Engineering, Sichuan University, Chengdu 610065, China

**Table of Contents**

1. Experimental details

2. Synthetic procedure and characterizations

3. Polymer Thermal Properties

4. Single crystal data

5. Electrostatic surface potential for polymers

6. OFET device fabrication

**1. Experimental details**

All commercially available reagents, solvents and chemicals were used without further purification unless otherwise mentioned. Anhydrous toluene was distilled from Na/benzophenone. ^1^H NMR and ^13^C NMR spectra were recorded using a Bruker Ascend 400 MHz spectrometer, the chemical shifts (δ) were referenced to residual protio-solvent signals. High-resolution mass spectra (HR-MS) were performed using ESI-TOF in positive mode. Polymer molecular weights (*M*_n_) were characterized on Polymer Laboratories GPC-PL220 high temperature GPC/SEC system (Agilent Technologies) at 150 ºC vs polystyrene standards using trichlorobenzene as the eluent. UV-vis absorption spectra were measured with a Shimadzu UV-3600 UV-VIS-NIR spectrophotometer. Cyclic voltammetry (CV) was performed under argon atmosphere using a CHI760E electrochemical workstation with tetra-*n*-butylammonium hexafluorophosphate (0.1 M) in acetonitrile as the supporting electrolyte. A glassy carbon working electrode, a Pt wire counter electrode, and a silver wire reference electrode were employed, the ferrocene/ferrocenium (Fc/Fc^+^) was used as the internal reference. Thermogravimetric analysis (TGA) curves were collected on Mettler STAR^e^ (TA Instrument) and differential scanning calorimetry (DSC) curves were recorded on Mettler STAR^e^ (TA Instrument) in nitrogen with a heating ramp of 10 ºC min^-1^.

**2. Synthetic procedure and characterizations**

**5,5ʹ-dibromo-[2,2ʹ-bipyridine] 1-oxide (BPyO):** In a 100 mL single-necked flask, BPy (2.0 g, 6.37 mmol) was dissolved in CHCl_3_ (35 mL), *m*-chloroperbenzoic acid (1.65 g, 9.56 mmol) was added and stirred at room temperature for 10 h. The mixture was extracted using DCM and water, the organic phase was dried with anhydrous Na_2_SO_4_. The crude product was purified by silica gel column chromatography with DCM: EA=1:1 as eluent to give BPyO. Yield: 1.87 g, 89%. ^1^H NMR (400 MHz, CDCl_3_): δ ppm 8.90 (d, *J* = 8.0 Hz, 1H), 8.76 (d, *J* = 4.0 Hz, 1H), 8.46 (d, *J* = 2.0 Hz, 1H), 8.16 (d, *J* = 8.0 Hz, 1H), 7.96 (dd, *J* = 8.0, 4.0 Hz, 1H), 7.49 (dd, *J* = 8.0, 4.0 Hz, 1H). ^13^C NMR (100 MHz, CDCl_3_): δ ppm 150.6, 147.1, 145.5, 142.0, 139.1, 128.9, 127.7, 126.4, 121.8, 120.1. HRMS (m/z): Calcd. for C_10_H_6_Br_2_N_2_O [M+H]^-^, Exact Mass: 328.8920; Found: 328.8924.

**5,5ʹ-dibromo-[2,2ʹ-bipyridine] 1,1ʹ-dioxide (BPyDO):** BPy (1.0 g, 3.18 mmol) and *m*-chloroperbenzoic acid (2.20 g, 12.72 mmol) were dissolved in CHCl_3_ (30 mL). The mixture was stirred at room temperature for 3d, then the solvent was removed under reduced pressure, the solid was dispersed into CH_3_OH and followed by filtration, BPyDO was obtained after recrystallization using DCM. Yield: 737 mg, 67%. ^1^H NMR (400 MHz, CDCl_3_): δ ppm 8.48 (d, *J* = 4.0 Hz, 2H), 7.61 (d, *J* = 12.0 Hz, 2H), 7.47 (dd, *J* = 8.0, 4.0 Hz, 2H). ^13^C NMR (100 MHz, CDCl_3_): δ ppm 141.5, 140.1, 128.4, 127.9, 121.5. HRMS (m/z): Calcd. for C_10_H_6_Br_2_N_2_O_2_ [M+H]^-^, Exact Mass: 344.8869; Found: 344.8876.

**General procedure for polymerizations of polymers.**

Two monomers (1.0 eq:1.0 eq), catalyst (0.015 eq) and ligand (0.12 eq) were added into a 10 mL glass tube and its content was pump/purge three times with argon. After added anhydrous toluene (3 mL) under argon flow, the tube was sealed with rubber cap. The mixture stirred at 120 ℃ for three days, then successively added 100 μL 2-(tributylstanny) thiophene and 100 μL 2-bromothiophene to react 0.5 h for stemming reaction. The mixture was dripped into 50 mL methanol and filtered, the solid was purified by Soxhlet extraction with methanol, acetone, hexane, dichloromethane and chloroform. The chloroform fraction was concentrated under reduced pressure and dripped into 50 mL methanol, the solid precipitate was filtered and dried under vacuum to afford polymers.


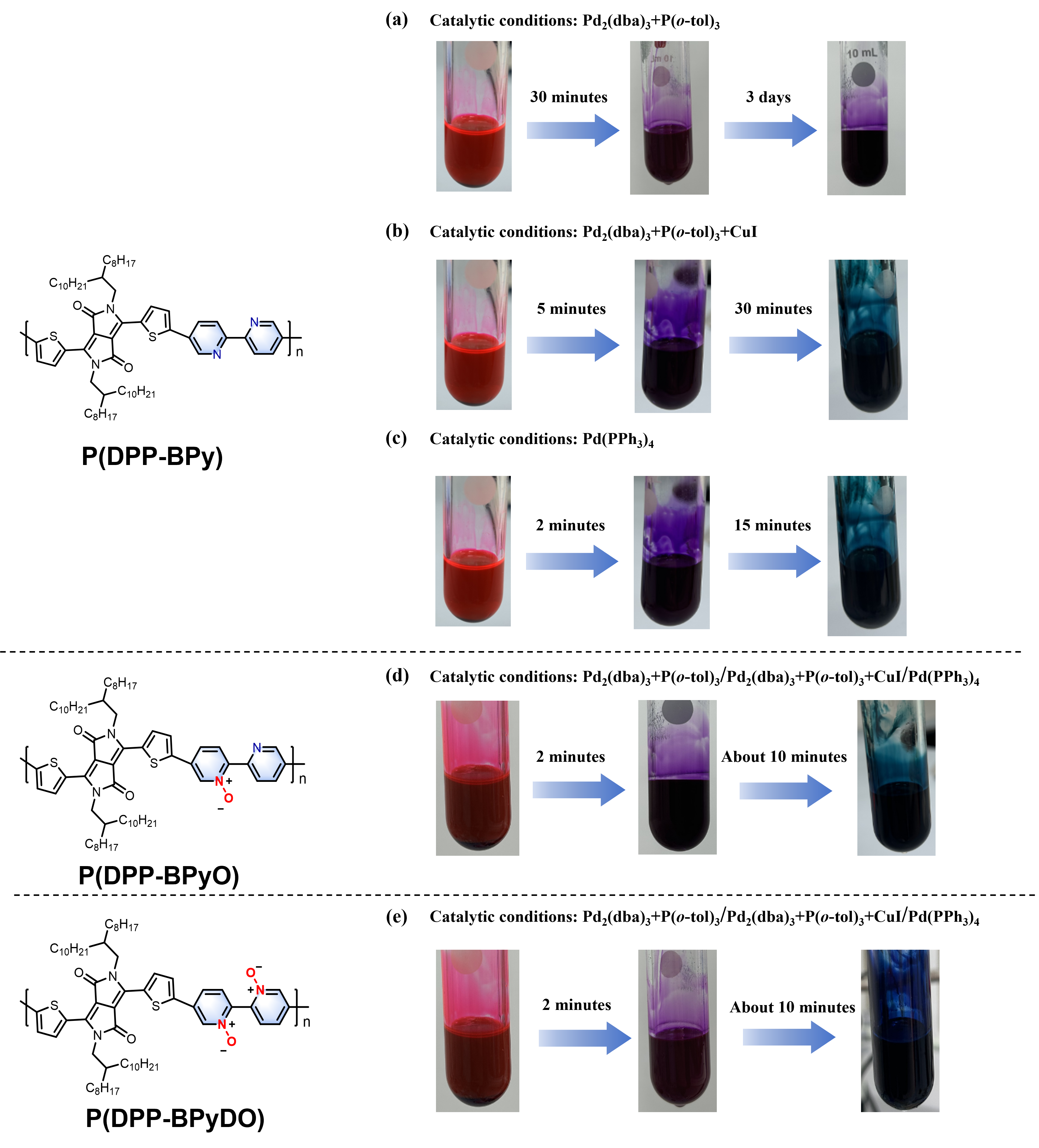


**Figure S1**. Different polymerization conditions for three polymers and their color variations.

P(DPP-BPy) cannot be obtained using this method.

Two monomers (1.0 eq each), Pd_2_(dba)_3_ (0.02 eq), P(*o*-tol)_3_ (0.16 eq) and CuI (0.1 eq) were added into a 10 mL glass tube. The glass tube was subjected to 3 pump/purge cycles with argon. After added anhydrous toluene (3 mL) under argon flow, the tube was sealed. The mixture was heated at 120 ℃ for 3 days and successively added 100 μL 2-(tributylstanny) thiophene and 100 μL 2-bromothiophene to react 30 min for stemming reaction. After cooling to room temperature, the mixture was dripped into 50 mL methanol and filtered, the solid was purified by Soxhlet extraction with methanol, acetone, hexane, dichloromethane and chloroform. The chloroform fraction was concentrated under reduced pressure and dripped into 50 mL methanol, the solid precipitate was filtered and dried under vacuum to afford P(DPP-BPy) with a yield of 39%. *M*_n_ = 8.0 kDa, PDI = 2.9.

Two monomers (1.0 eq each) and Pd(PPh_3_)_4_ (0.02 eq) were added into a 10 mL glass tube. The glass tube was subjected to 3 pump/purge cycles with argon. After added anhydrous toluene (3 mL) under argon flow, the tube was sealed. The mixture was heated at 120 ℃ for 3 days and successively added 100 *μ*L 2-(tributylstanny) thiophene and 100 *μ*L 2-bromothiophene to react 30 min for stemming reaction. After cooling to room temperature, the mixture was dripped into 50 mL methanol and filtered, the solid was purified by Soxhlet extraction with methanol, acetone, hexane, dichloromethane and chloroform. The chloroform fraction was concentrated under reduced pressure and dripped into 50 mL methanol, the solid precipitate was filtered and dried under vacuum to afford P(DPP-BPy) with a yield of 58%. *M*_n_ = 22.5 kDa, PDI = 1.5.

Two monomers (1.0 eq each), Pd_2_(dba)_3_ (0.02 eq) and P(*o*-tol)_3_ (0.16 eq) were added into a 10 mL glass tube. The glass tube was subjected to 3 pump/purge cycles with argon. After added anhydrous toluene (3 mL) under argon flow, the tube was sealed. The mixture was heated at 120 ℃ for 37 min and stopped immediately to avoid insolubility. After cooling to room temperature, the mixture was dripped into 50 mL methanol and filtered, the solid was purified by Soxhlet extraction with methanol, acetone, hexane, dichloromethane and chloroform. The chloroform fraction was concentrated under reduced pressure and dripped into 50 mL methanol, the solid precipitate was filtered and dried under vacuum to afford P(DPP-BPyO) with a yield of 79%. *M*_n_ = 49.7 kDa, PDI = 2.1.

 Two monomers (1.0 eq each), Pd_2_(dba)_3_ (0.02 eq), P(*o*-tol)_3_ (0.16 eq) and CuI (0.1 eq) were added into a 10 mL glass tube. The glass tube was subjected to 3 pump/purge cycles with argon. After added anhydrous toluene (3 mL) under argon flow, the tube was sealed. The mixture was heated at 120 ℃ for 10 h and stopped immediately. After cooling to room temperature, the mixture was dripped into 50 mL methanol and filtered, the solid was purified by Soxhlet extraction with methanol, acetone, hexane, dichloromethane and chloroform. The chloroform fraction was concentrated under reduced pressure and dripped into 50 mL methanol, the solid precipitate was filtered and dried under vacuum to afford P(DPP-BPyO) with a yield of 74%. *M*_n_ = 33.4 kDa, PDI = 1.8.

Two monomers (1.0 eq each) and Pd(PPh_3_)_4_ (0.02 eq) were added into a 10 mL glass tube. The glass tube was subjected to 3 pump/purge cycles with argon. After added anhydrous toluene (3 mL) under argon flow, the tube was sealed. The mixture was heated at 120 ℃ for 3 days and successively added 100 μL 2-(tributylstanny) thiophene and 100 μL 2-bromothiophene to react 30 min for stemming reaction. After cooling to room temperature, the mixture was dripped into 50 mL methanol and filtered, the solid was purified by Soxhlet extraction with methanol, acetone, hexane, dichloromethane and chloroform. The chloroform fraction was concentrated under reduced pressure and dripped into 50 mL methanol, the solid precipitate was filtered and dried under vacuum to afford P(DPP-BPyO) with a yield of 70%. *M*_n_ = 50.9 kDa, PDI = 2.6.

Two monomers (1.0 eq each), Pd_2_(dba)_3_ (0.02 eq) and P(*o*-tol)_3_ (0.16 eq) were added into a 10 mL glass tube. The glass tube was subjected to 3 pump/purge cycles with argon. After added anhydrous toluene (3 mL) under argon flow, the tube was sealed. The mixture was heated at 120 ℃ for 3 days and successively added 100 μL 2-(tributylstanny) thiophene and 100 μL 2-bromothiophene to react 30 min for stemming reaction. After cooling to room temperature, the mixture was dripped into 50 mL methanol and filtered, the solid was purified by Soxhlet extraction with methanol, acetone, hexane, dichloromethane and chloroform. The chloroform fraction was concentrated under reduced pressure and dripped into 50 mL methanol, the solid precipitate was filtered and dried under vacuum to afford P(DPP-BPyDO) with a yield of 75%. *M*_n_ = 53.3 kDa, PDI = 1.8.

Two monomers (1.0 eq each), Pd_2_(dba)_3_ (0.02 eq), P(*o*-tol)_3_ (0.16 eq) and CuI (0.1 eq) were added into a 10 mL glass tube. The glass tube was subjected to 3 pump/purge cycles with argon. After added anhydrous toluene (3 mL) under argon flow, the tube was sealed. The mixture was heated at 120 ℃ for 3 days and successively added 100 μL 2-(tributylstanny) thiophene and 100 μL 2-bromothiophene to react 30 min for stemming reaction. After cooling to room temperature, the mixture was dripped into 50 mL methanol and filtered, the solid was purified by Soxhlet extraction with methanol, acetone, hexane, dichloromethane and chloroform. The chloroform fraction was concentrated under reduced pressure and dripped into 50 mL methanol, the solid precipitate was filtered and dried under vacuum to afford P(DPP-BPyDO) with a yield of 66%. *M*_n_ = 30.0 kDa, PDI = 1.7.

Two monomers (1.0 eq each) and Pd(PPh_3_)_4_ (0.1 eq) were added into a 10 mL glass tube. The glass tube was subjected to 3 pump/purge cycles with argon. After added anhydrous toluene (3 mL) under argon flow, the tube was sealed. The mixture was heated at 120 ℃ for 3 days and successively added 100 μL 2-(tributylstanny) thiophene and 100 μL 2-bromothiophene to react 30 min for stemming reaction. After cooling to room temperature, the mixture was dripped into 50 mL methanol and filtered, the solid was purified by Soxhlet extraction with methanol, acetone, hexane, dichloromethane and chloroform. The chloroform fraction was concentrated under reduced pressure and dripped into 50 mL methanol, the solid precipitate was filtered and dried under vacuum to afford P(DPP-BPyDO) with a yield of 68%. *M*_n_ = 35.0 kDa, PDI = 1.6.

**Figure S2**. ^1^H NMR spectrum of compound **BPyO** (400MHz, r.t., in CDCl_3_).


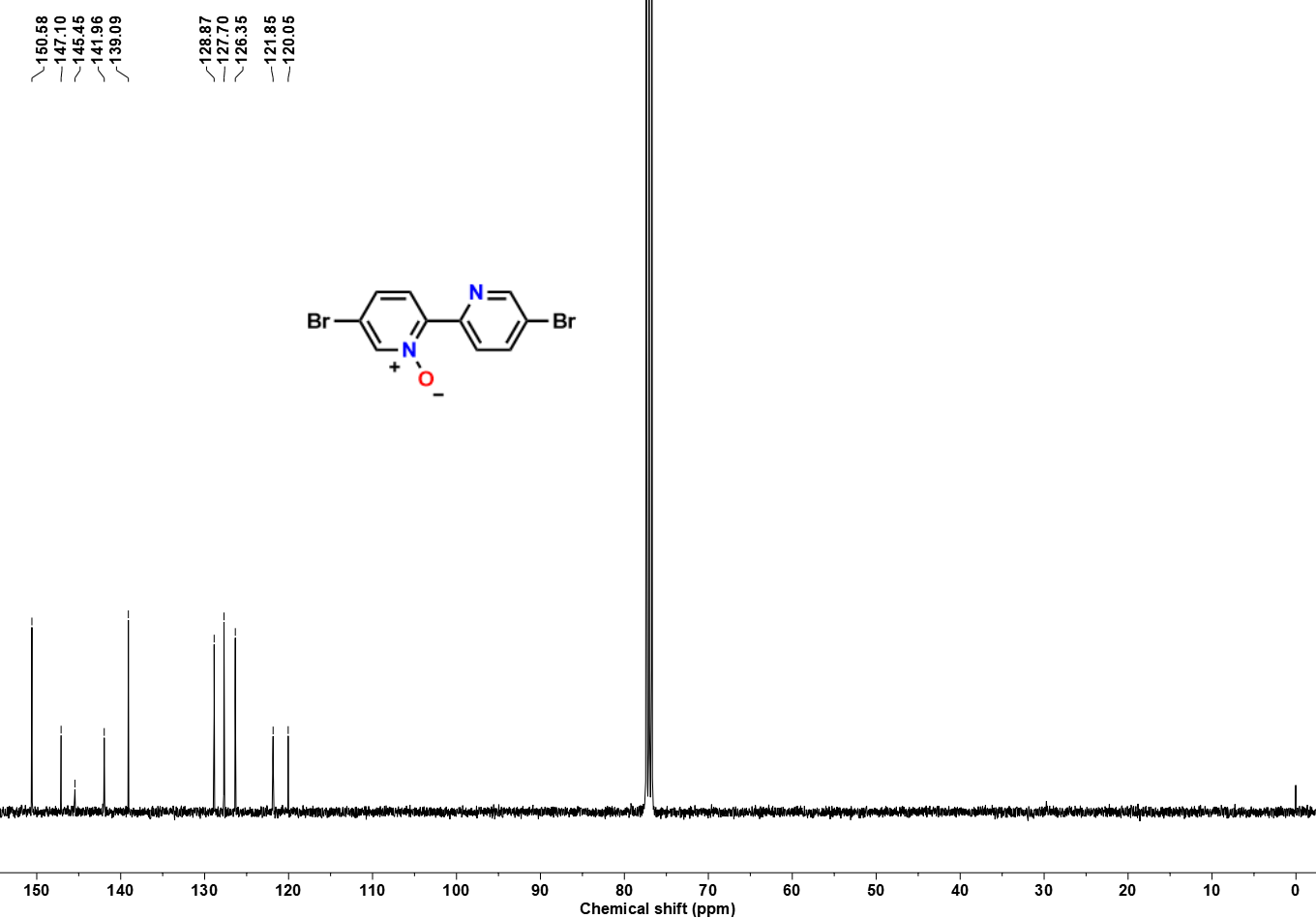


**Figure S3**. ^13^C NMR spectrum of compound **BPyO** (100MHz, r.t., in CDCl_3_).

**Figure S4**. The HRMS [M+H]^-^ of **BPyO**.

**Figure S5**. ^1^H NMR spectrum of compound **BPyDO** (400MHz, r.t., in CDCl_3_).

**Figure S6**. ^13^C NMR spectrum of compound **BPyDO** (100MHz, r.t., in CDCl_3_).

**Figure S7**. The HRMS [M+H]^-^ of **BPyDO**.


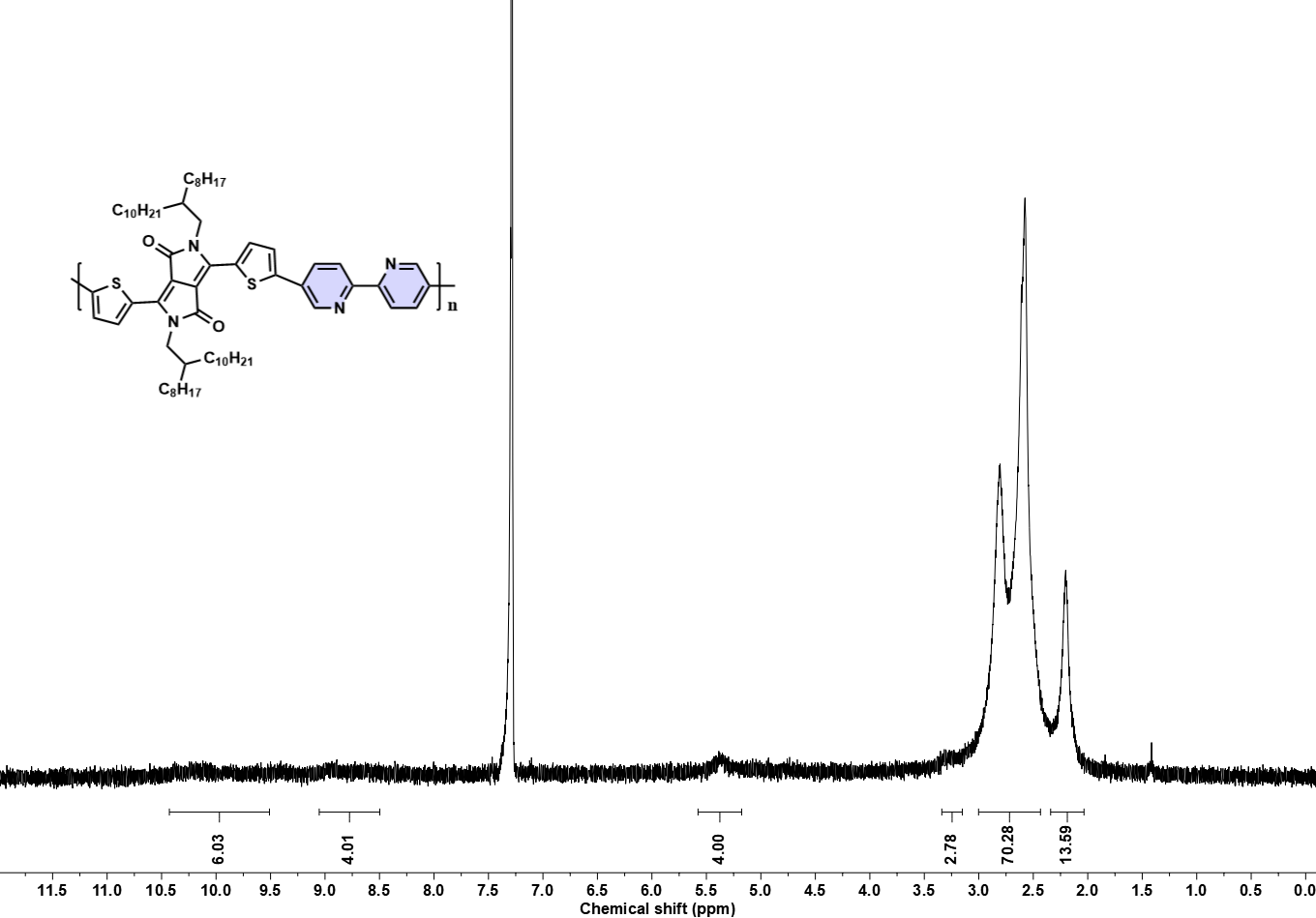


**Figure S8**. ^1^H NMR spectrum of **P(DPP-BPy)** (500 MHz, 80 ℃, in C_2_D_2_Cl_4_).


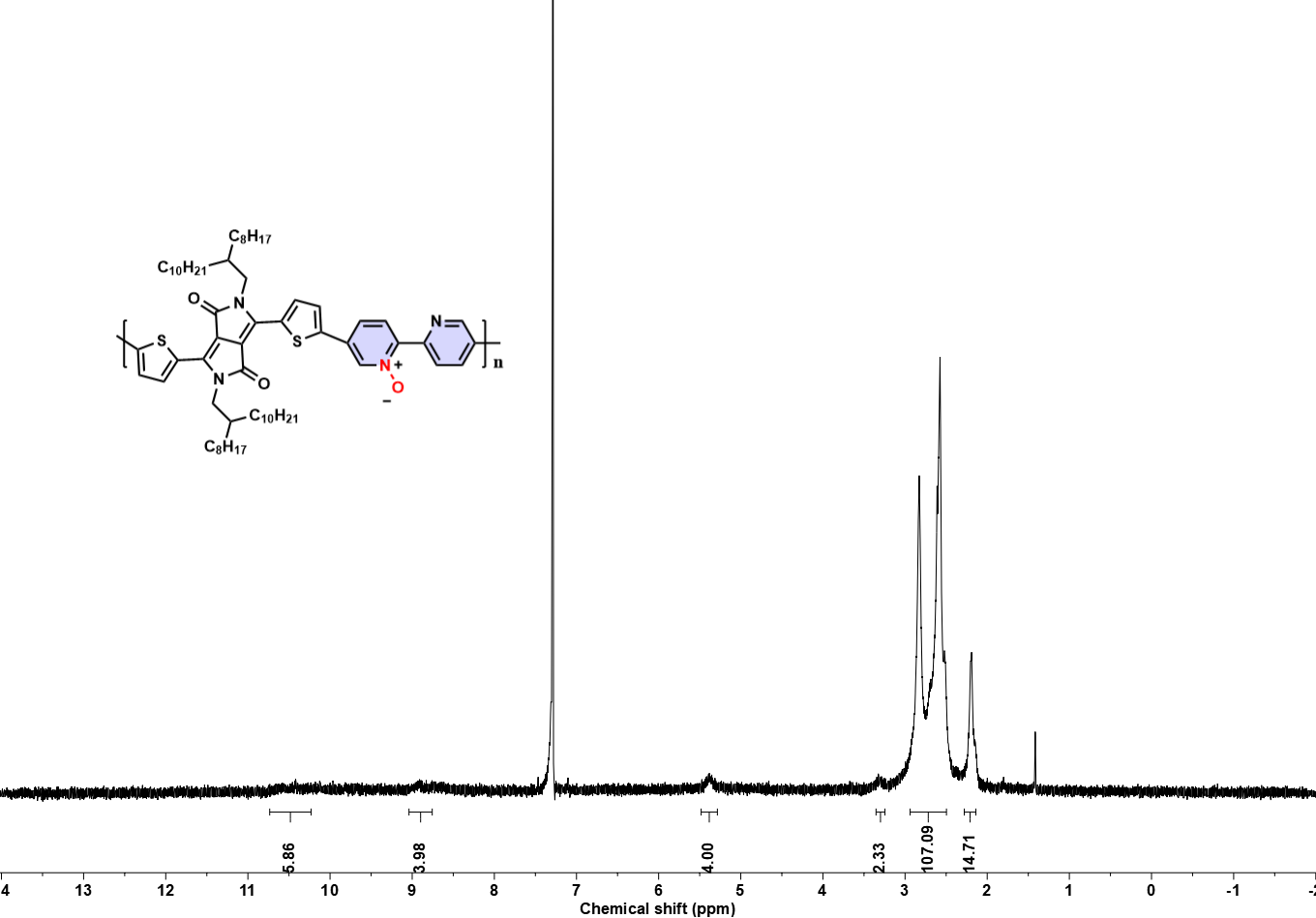


**Figure S9**. ^1^H NMR spectrum of **P(DPP-BPyO)** (500 MHz, 80 ℃, in C_2_D_2_Cl_4_).


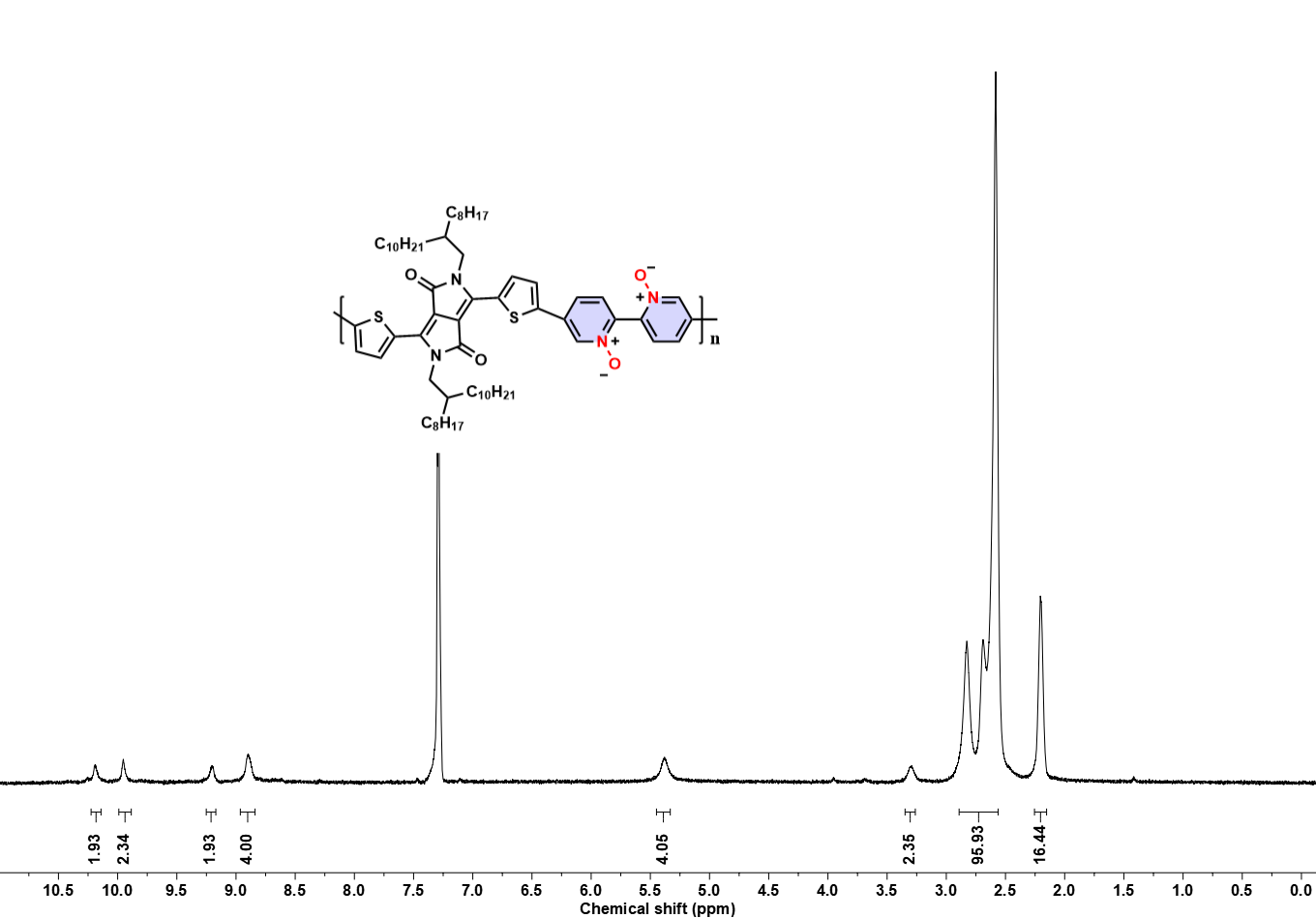


**Figure S10**. ^1^H NMR spectrum of **P(DPP-BPyDO)** (500 MHz, 80 ℃, in C_2_D_2_Cl_4_).

**3. Polymer thermal properties**


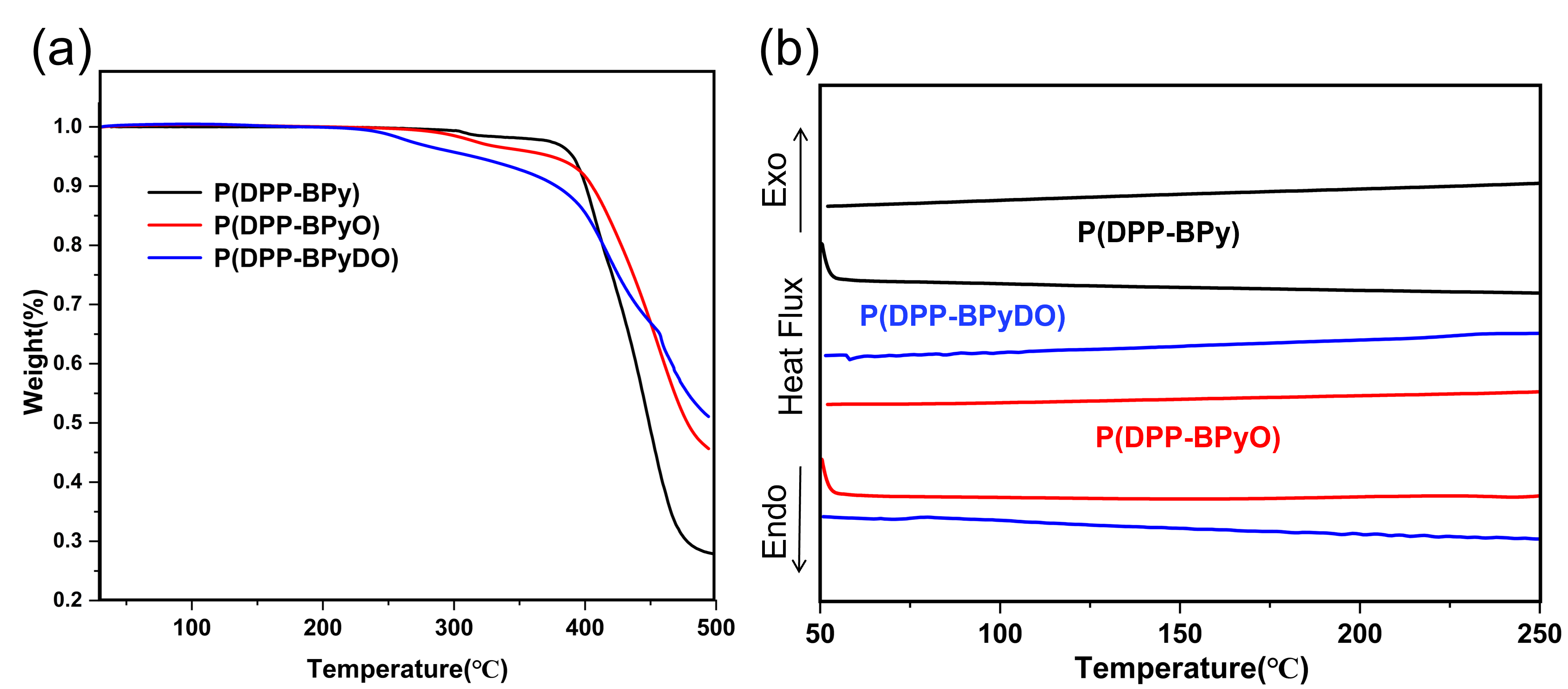


**Figure S11**. (**a**) Thermogravimetric analysis of P(DPP-BPy), P(DPP-BPyO) and P(DPP-BPyDO) at a heating rate of 10 ℃ min^-1^. (**b**) DSC thermograms of polymers P(DPP-BPy), P(DPP-BPyO) and P(DPP-BPyDO). The DSC curves are from the second heating and first cooling scans with a ramp rate of 10 ℃ min^-1^. N_2_ was used as the purge gas for both TGA and DSC measurements.

**4. Single crystal data**

Single crystals of BPyO and BPyDO were prepared by slow evaporation of dichloromethane solution at room temperature. X-ray crystallography was carried out on a Bruker SMART APEX-II CCD diffractometer with graphite monochromated Mo-Kα radiation at 296 K.

**Table S1**. Crystallographic data for **BPyO** and **BPyDO** single crystals.

| **Compound** | **BPyO** | **BPyDO** |
| --- | --- | --- |
| Empirical formula | C_10_H_6_Br_2_N_2_O | C_10_H_6_Br_2_N_2_O_2_ |
| Formula weight (g mol^-1^) | 329.99 | 345.99 |
| Crystal color | colourless | colourless |
| Crystal system | Monoclinic | Monoclinic |
| Space group | P21/c | C2/c |
| a, (Å) | 10.275(4) | 19.565(5) |
| b, (Å) | 3.9087(9) | 3.9113(6) |
| c, (Å) | 13.378(5) | 14.492(3) |
| α, (deg.) | 90 | 90 |
| β, (deg.) | 101.617(11) | 106.053(6) |
| γ, (deg.) | 90 | 90 |
| Volume, (Å3) | 526.3(3) | 1065.8(4) |
| Z | 2 | 4 |
| Density, (g cm^-3^)  hmax, kmax, lmax | 2.082  13, 4, 16 | 2.156  23, 4, 18 |
| CCDC NO. | 2367453 | 2367454 |

1. **Electrostatic surface potential for polymers**

**
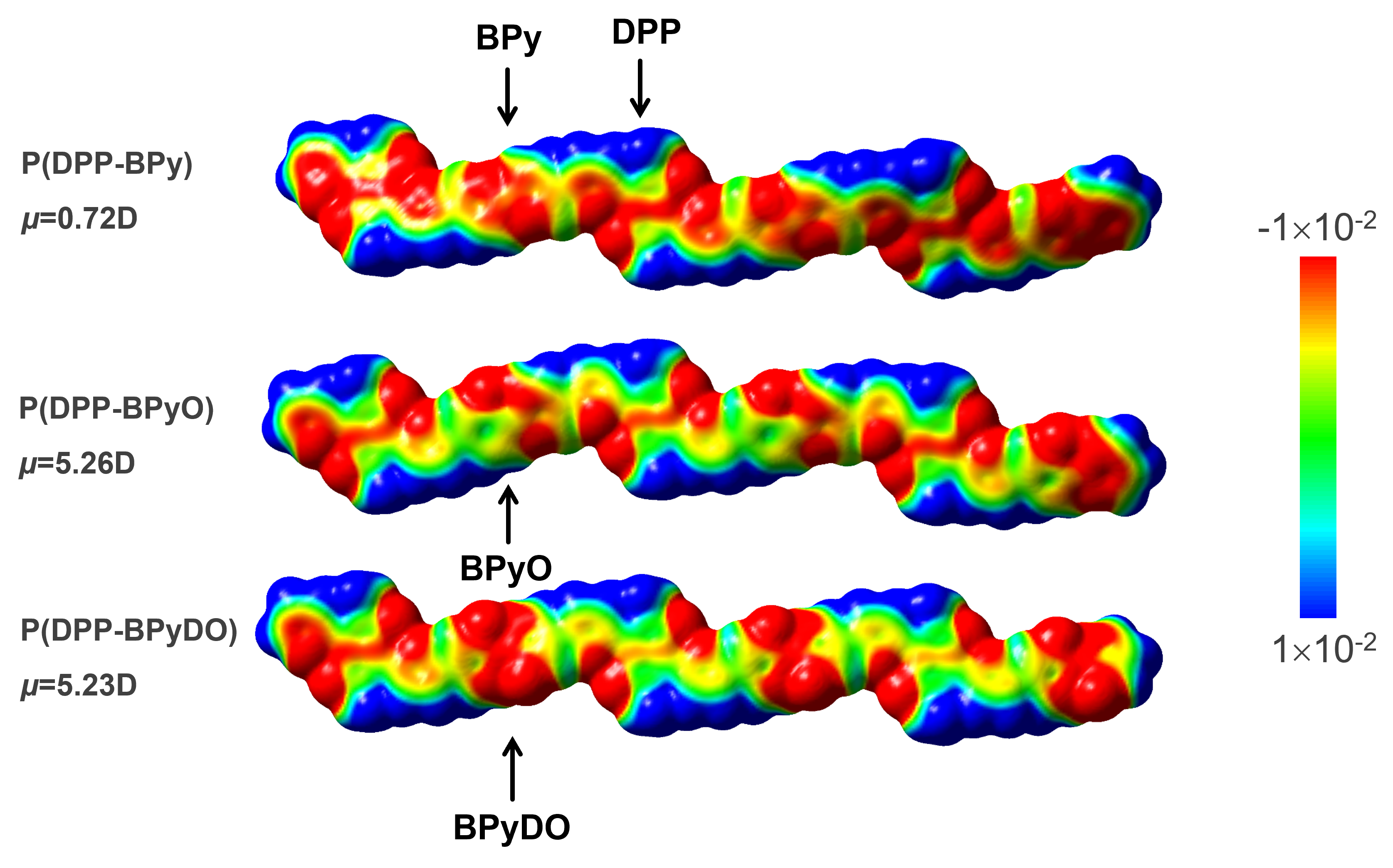
**

**Figure S12.** Electrostatic surface potential of P(DPP-BPy), P(DPP-BPyO) and P(DPP-BPyDO).

**6. OFET device fabrication**

The top-gate bottom-contact (TGBC) organic field-effect transistors are fabricated to evaluate the charge transport performance of three polymers P(DPP-BPy), P(DPP-BPyO) and P(DPP-BPyDO) under different polymerization conditions. 30 nm Au were deposited on a cleaned glass plate, then the substrate was successively transferred into deionized water, acetone and isopropanol for sonication. After that, the substrate was transferred into glove box with nitrogen atmosphere, chloroform solution of polymers was spin-coated with 2000 rpm and annealed at 100 ℃ for 30 min. The dielectric layer was spin-coated onto the polymer semiconductor layer using a 60 μL polymethyl methacrylate (PMMA) butylacetate solution and annealed at 100 ℃ for 30 minutes. Finally, a 100 nm Ag gate electrode was thermally evaporated on the the dielectric layer.

The saturation charge-carrier mobility (*μ*) was calculated according to the equation:

*I*_DS_ = (W*μ*C_i_ /2L)(*V*_G_-*V*_th_)^2^

where the *I*_DS_ is the source-drain current, *V*_G_ and *V*_th_ are the gate voltage and threshold, respectively. *C*_i_ is the capacitance per unit area of dielectric and *C*_i_ (PMMA) = 3 nF·cm^−2^.

Transistors performances of **P(DPP-BPy)**, **P(DPP-BPyO)** and **P(DPP-BPyDO)** under different polymerization conditions:


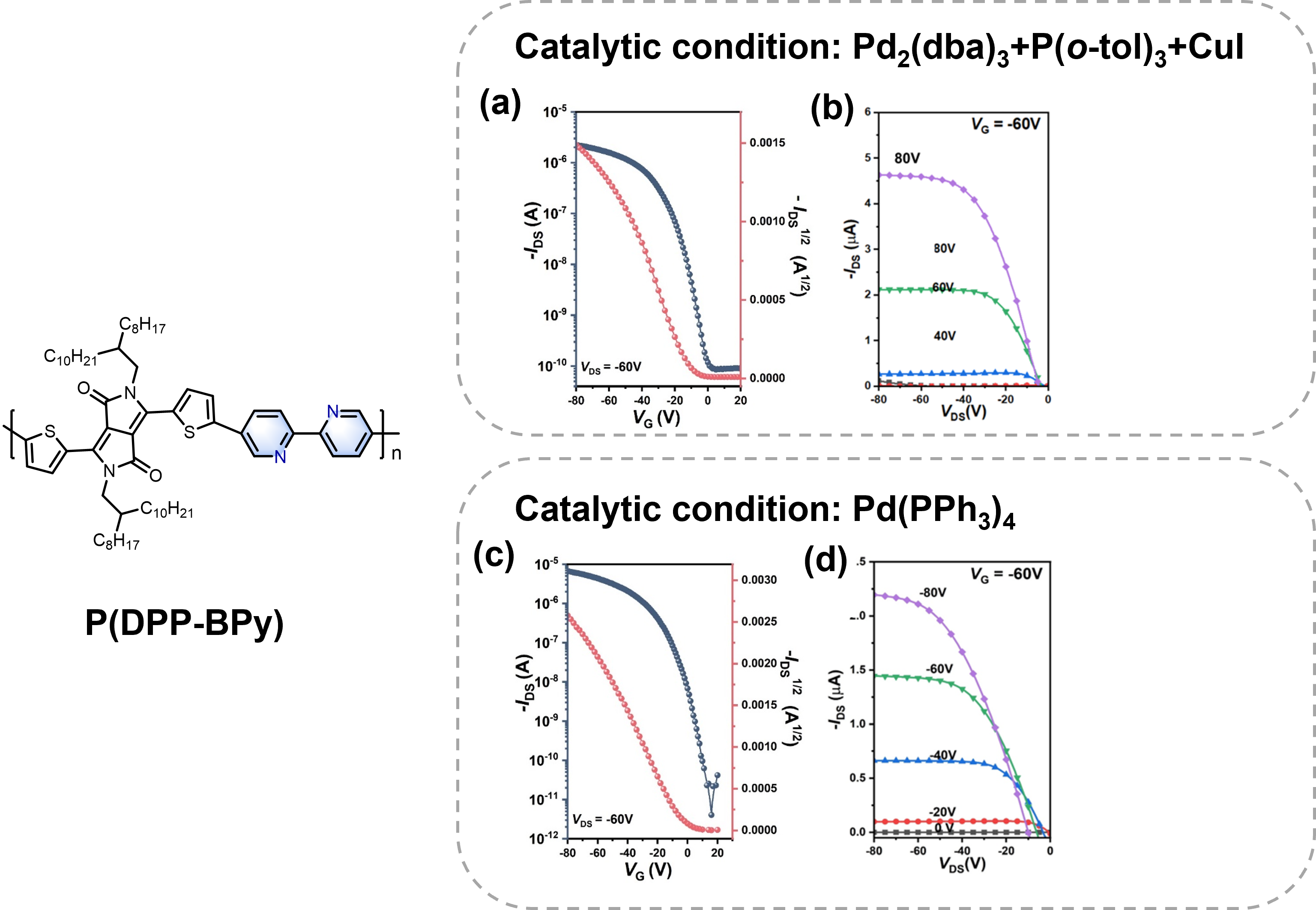


**Figure S13.** The transfer (a, c) and output curves (b, d) of **P(DPP-BPy)** under different polymerization conditions.


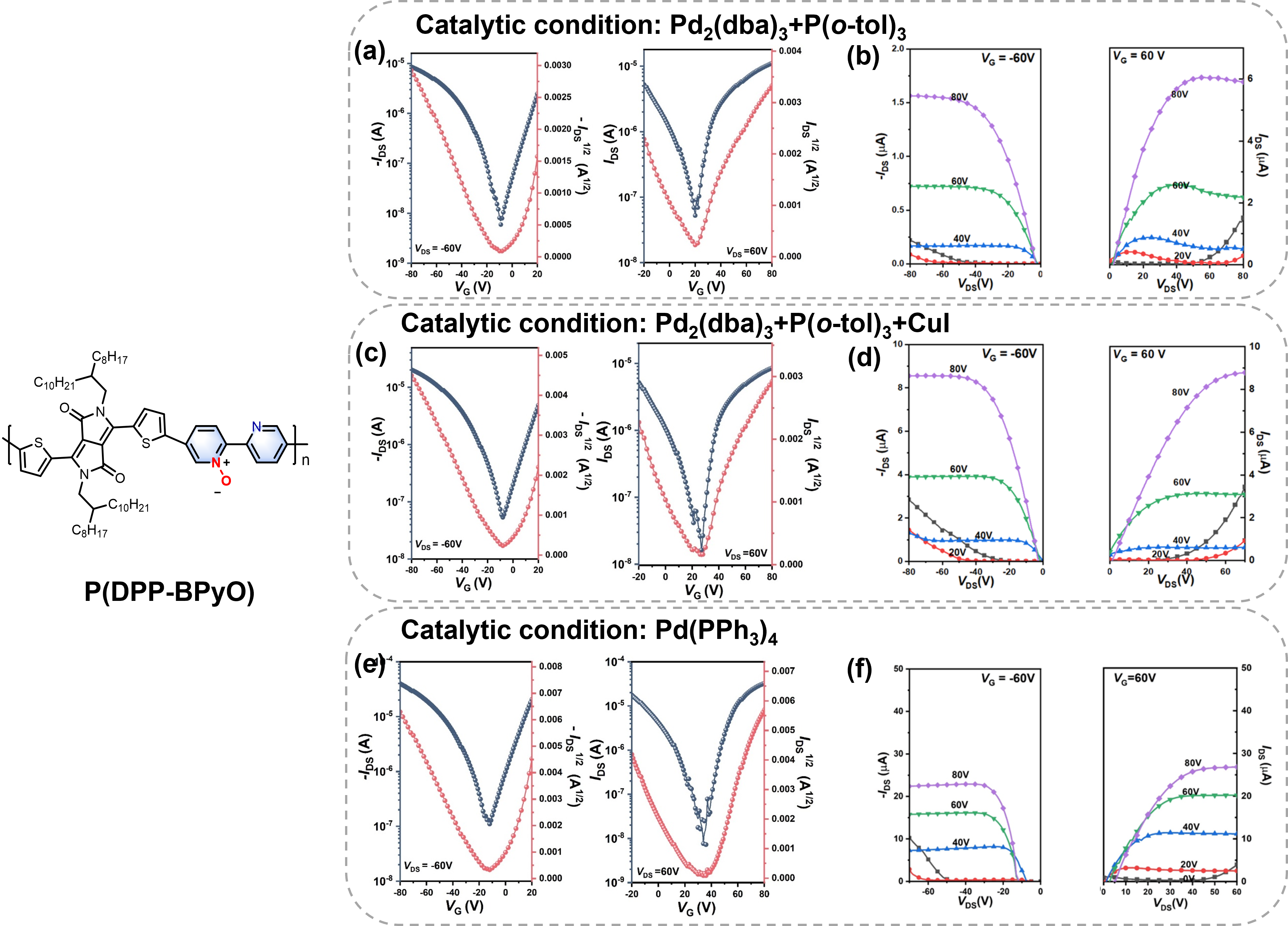


**Figure S14.** The transfer (a, c, e) and output curves (b, d, f) of **P(DPP-BPyO)** under different polymerization conditions.


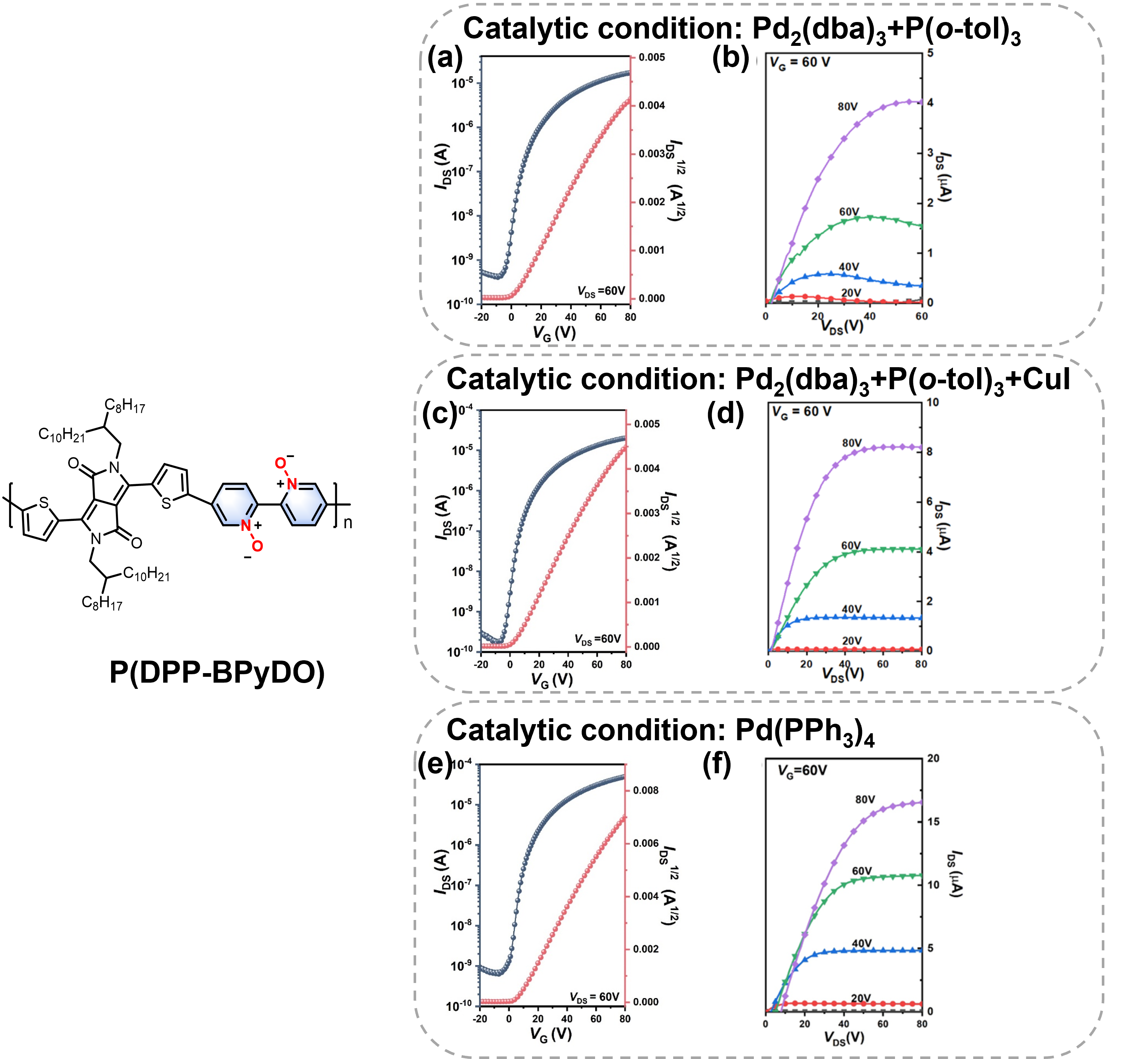


**Figure S15.** The transfer (a, c, e) and output curves (b, d, f) of **P(DPP-BPyDO)** under different polymerization conditions.

**Table S2.** Transistors performances of polymers synthesized under different conditions

| Polymer  (Catalysis) | *μ*_h_  (cm^2^V^−1^s^−1^) | *V*_h,onset_  (V) | *I*_on_/*I*_off_ | *μ*_e_  (cm^2^V^−1^s^−1^) | *V*_e,onset_ (V) | *I*_on_/*I*_off_ |
| --- | --- | --- | --- | --- | --- | --- |
| P(DPP-BPy) (Pd_2_(dba)_3_+CuI) | 0.010  (0.008) | -10 to -15 | 10^4^～10^5^ | NA | NA | NA |
| P(DPP-BPyO) (Pd_2_(dba)_3_+CuI) | 0.072  (0.053) | -10 to -20 | 10^2^～10^3^ | 0.093  (0.062) | 20 to 30 | 10^2^～10^3^ |
| P(DPP-BPyDO) (Pd_2_(dba)_3_+CuI) | NA | NA | NA | 0.067  (0.032) | 5 to 10 | 10^4^～10^5^ |
| P(DPP-BPy) (Pd_2_(dba)_3_) | NA | NA | NA | NA | NA | NA |
| P(DPP-BPyO) (Pd_2_(dba)_3_) | 0.035  (0.023) | -10 to -20 | 10^2^～10^3^ | 0.084  (0.058) | 20 to 30 | 10^2^～10^3^ |
| P(DPP-BPyDO) (Pd_2_(dba)_3_) | NA | NA | NA | 0.056  (0.030) | 5 to 10 | 10^4^～10^5^ |

*μ*_h_ and *μ*_e_ were maximum mobilities, the average values are in parentheses (>10 devices).


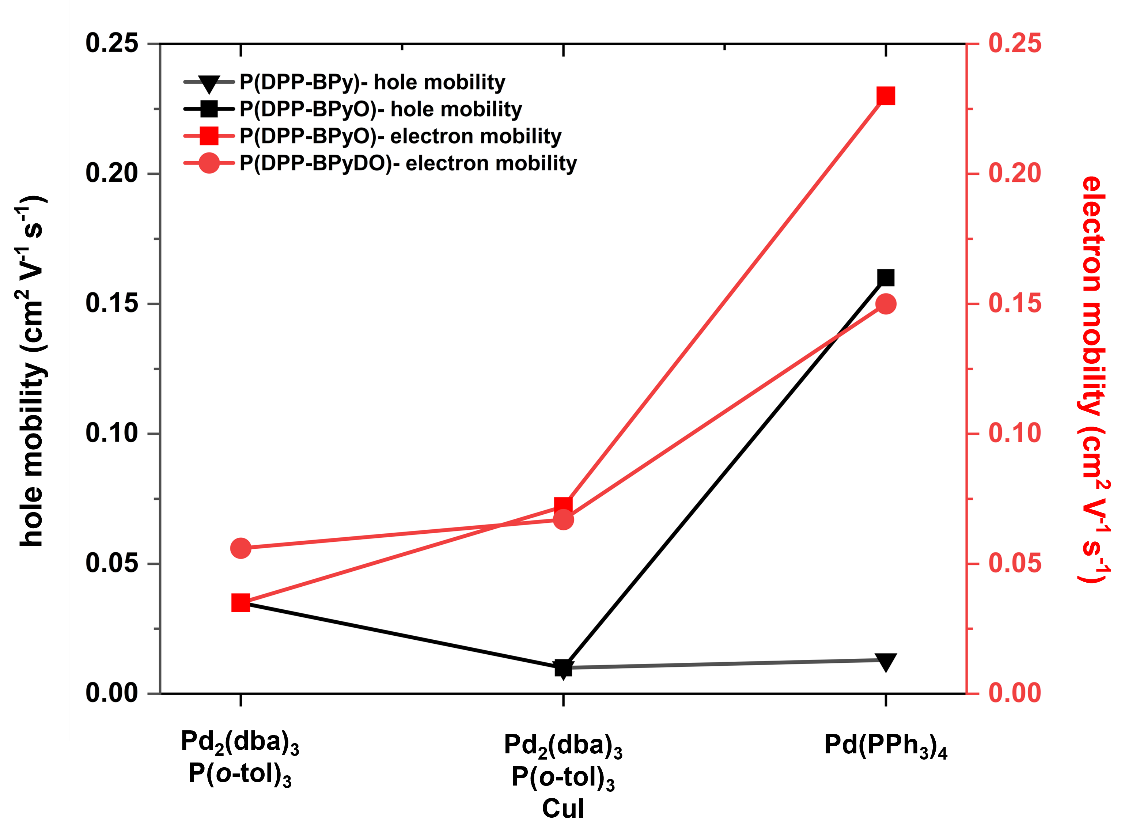


**Figure S16.** The charge carrier mobility of polymers obtained under different catalytic condition.

**
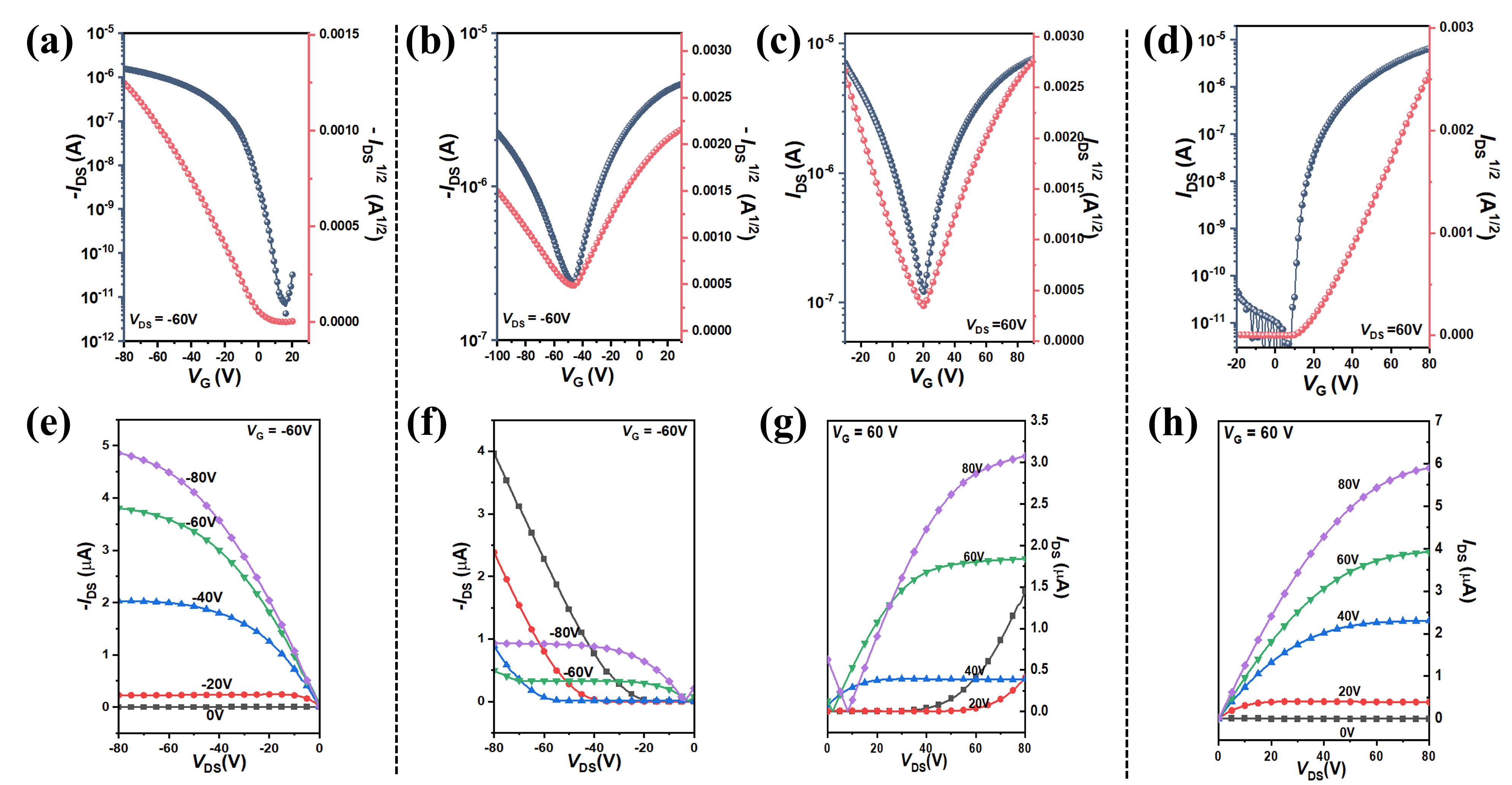
**

**Figure S17.** p-type transfer (a and b) and output characteristics (e and f) of pristine P(DPP-BPy) and P(DPP-BPyO). n-type transfer (c and d) and output characteristics (g and h) of pristine P(DPP-BPyO) and P(DPP-BPyDO).

**Table S3.** Transistors performances of polymers prepared before and after thermal annealing.

| Polymer | | Thermal process | p-Type | | | n-Type | | |
| --- | --- | --- | --- | --- | --- | --- | --- | --- |
|  |  |  | *μ*_h_^[a]^  (cm ^2^ V^-1^ s^-1^) | *I*_on_/*I*_off_ | *V*_th_ | *μ*_e_^[a]^  (cm ^2^ V^-1^ s^-1^) | *I*_on_/*I*_off_ | *V*_th_ |
| P(DPP-BPy) | Without annealing | | 2.1×10^−3^(1.6×10^−3^) | 10^4^~10^5^ | 0~-5 | NA | NA | NA |
|  | After annealing | | 1.3×10^−2^ (10^−2^) | 10^4^~10^5^ | -5~-10 | NA | NA | NA |
| P(DPP-BPyO) | Without annealing | | 5.8×10^−3^(3.9×10^−3^) | 10^1^~10^2^ | -40~-50 | 1.5×10^−2^(1.1×10^−2^) | 10^2^~10^3^ | 20~30 |
|  | After annealing | | 1.6 ×10^−1^(7.7×10^−2^) | 10^2^~10^3^ | -15~-20 | 2.3×10^−1^ (9.5×10^−2^) | 10^2^~10^3^ | 30~45 |
| P(DPP-BPyDO) | Without annealing | | NA | NA | NA | 1.2×10^−2^(9.2×10^−3^) | 10^4^~10^5^ | 10~15 |
|  | After annealing | | NA | NA | NA | 0.15 (7.1×10^−2^) | 10^4^~10^5^ | 5~10 |

^[a]^ maximum mobilities; the average values are in parentheses (>10 devices).

**Table S4**. Packing parameters for these polymers P(DPP-BPy), P(DPP-BPyO) and P(DPP-BPyDO) based on the GIWAXS measurements.

| Polymer | OOP | | | IP | | |
| --- | --- | --- | --- | --- | --- | --- |
|  | (010) | | | (100) | | |
|  | q  (Å) | d-spacing  (Å) | CCL  (Å) | q  (Å) | d-spacing  (Å) | CCL  (Å) |
| P(DPP-BPy) | 1.703 | 3.686 | 106.243 | 0.310 | 20.268 | 91.303 |
| P(DPP-BPyO) | 1.692 | 3.713 | 142.521 | 0.311 | 20.203 | 73.060 |
| P(DPP-BPyDO) | 1.383 | 4.543 | 12.459 | 0.301 | 20.874 | 47.124 |
